# Supplementary material for: Uncovering the complex relationship between balding, testosterone and skin cancers in men
Source: Nat Commun. 2023 Oct 3;14:5962. doi: 10.1038/s41467-023-41231-8 (PMC10547720; doi:10.1038/s41467-023-41231-8)
Supplement: Supplementary file 1 — Supplementary Information [file 41467_2023_41231_MOESM1_ESM.pdf]

Supplementary Information for manuscript

# Uncovering the complex relationship between balding and skin cancers in men

## Table of Contents

Supplementary Notes 4

Supplementary Notes 1. Study descriptions 4

Supplementary Notes 2. Definition of MPB in the UK Biobank 6

Supplementary Notes 3. Derivation of free testosterone in the UK Biobank 6

Supplementary Notes 4. Estimation of genetic association with MPB and endogenous sex-hormones from non-overlapping UKB subsample for use in MR analyses 7

Supplementary Notes 5. Definition of site-specific skin cancers evaluated 7

Supplementary Notes 6. Power calculation for MR analysis 7

Supplementary Notes 7. Estimates derived from alternative MR models 8

Supplementary Notes 8. Selection of pleiotropic variants for PheWAS analyses and validation via univariate MR analysis on MPB 9

Supplementary Notes 9. Curation of instrument and assessment of instrument strength for MVMR analysis 10

Supplementary Tables 11

Supplementary Table 1. Description of cases and controls for individual studies involved in the GenoMEL Phase 2 GWAS meta-analysis. 11

Supplementary Table 2. Distribution of cases and controls for analyses on major body-site categories 12

Supplementary Table 3. Body-site specific sample size distribution for melanoma and keratinocyte cancer cases in the UK Biobank 14

Supplementary Table 4. Body-site specific sample size distribution for melanoma and keratinocyte cancer cases in the Australian (QSKIN and MIA) datasets 15

Supplementary Table 5. Conditional F-statistics to measure instrument strength for multivariable MR in various combination of traits 17

Supplementary Table 6. Estimated univariate MR derived ORs for per 1 SD change increase in genetically predicted MPB score on skin cancer risk 18

Supplementary Table 7. Estimated univariate MR derived ORs for per 1 SD change increase in genetically predicted free testosterone on skin cancer risk 20

Supplementary Table 8. Estimated univariate MR derived ORs for per 1 SD change increase in genetically predicted total testosterone on skin cancer risk 22

Supplementary Table 9. Estimated univariate MR derived ORs for per 1 SD change increase in genetically predicted SHBG on skin cancer risk 24
Supplementary Table 10. Estimated Multivariable MR derived marginal OR on skin cancer risk from MVMR model including totalT, freeT and MPB 26
Supplementary Table 11. PheWAS summary on detected MR-PRESSO SNP outliers on a range of skin-related risk factors and disease traits 27
Supplementary Table 12. Univariate MR association between skin/hair colour and MPB score (incl alt models) 31
Supplementary Table 13. Estimated Multivariable MR derived marginal OR on skin cancer risk from MVMR model including totalT, freeT, hair colour and MPB 32
Supplementary Table 14. Estimated Multivariable MR derived marginal OR on skin cancer risk from MVMR model including totalT, freeT, hair colour, skin colour and MPB 33
Supplementary Table 15. Sensitivity analyses assessing the influence of the 4 detected MR-PRESSO SNP outliers on MVMR marginal OR estimates 34
Supplementary Table 16. Estimated heterogeneity among SNP estimates on MPB and skin cancer 35
Supplementary Table 17. Estimated heterogeneity among SNP estimates on MPB and skin cancer using MPB variants with p<1e-5 in the independent UKB subset. 36
Supplementary Table 18. Estimated MR ORs for per 1 SD change increase in genetically predicted MPB score on skin cancer risk after removal of (n=4) pleiotropic SNP outliers 37
Supplementary Table 19. Estimated (meta-analysed) MR ORs for per 1 SD change increase in genetically predicted MPB score on skin cancer risk stratified by major body site categories. 39
Supplementary Table 20. Estimated MR ORs for per 1 SD change increase in genetically predicted MPB score on cutaneous melanoma risk stratified by (thick vs thin) Breslow thickness in the MIA dataset. 42
Supplementary Figures 43
Supplementary Figure 1. The anatomical definition for body site categories used in the present analyses 43
Supplementary Figure 2. Comparison of SNP effect sizes between the Ruth et al total testosterone GWAS and those derived from the UK Biobank (non-overlapping) subset. 44
Supplementary Figure 3. Comparison of SNP effect sizes between the Ruth et al free testosterone GWAS and those derived from the UK Biobank (non-overlapping) subset. 45
Supplementary Figure 4. Comparison of SNP effect sizes between the Ruth et al SHBG GWAS and those derived from the UK Biobank (non-overlapping) subset. 46
Supplementary Figure 5. Estimated statistical power for MR analysis to detect plausible OR effect sizes in our present analysis. 47
Supplementary Figure 6. MR funnel plots for the univariable MR association between MPB and BCC in the UK Biobank and QSKIN datasets 48

Supplementary Figure 7. MR funnel plots for the univariable MR association between MPB and SCC in the UK Biobank and QSKIN datasets 49

Supplementary Figure 8. MR funnel plots for the univariable MR association between MPB and all KC in the UK Biobank and QSKIN datasets 50

Supplementary Figure 9. MR funnel plots for the univariable MR association between MPB and melanoma 51

Supplementary Figure 10. PheWAS plot for rs2669871 52

Supplementary Figure 11. PheWAS plot for rs3847069 52

Supplementary Figure 12. PheWAS plot for rs1805007 53

Supplementary Figure 13. PheWAS plot for rs12203592 53

Reference 54

# Supplementary Notes

## Supplementary Notes 1. Study descriptions

### GenoMEL

The Melanoma Genetics Consortium (GenoMEL) is the world's leading consortium for familial melanoma research. Participating studies from the GenoMEL were predominantly from populations of European ancestry, with the largest contributing study from Australia (by the Melanoma Institute of Australia). We used the GenoMEL GWAS summary data on melanoma susceptibility performed only on male participants for the present study. Details of the curation of phenotype and sample size contribution from participating studies involved in the Landi et al. GWAS analysis [1] had been previously provided. For study specific acknowledgement and funding information, please also see the supplementary information section in Landi et al. [1].

### QSKIN cohort

The QSkin Sun and Health study is a prospective cohort study, with the first wave of recruitment collecting extensive health questionnaires (including self-report pigmentation and sun-protective behaviours), and medical record data linkage, for 43,794 participants between 2011 and 2016. All participants were recruited with informed consent, and ethical approval was managed by the Human Research Ethics committee of QIMR Berghofer Medical Research Institute. Melanoma and KC status was confirmed through a combination of linkage to cancer registry, pathology databases, and Australian Medicare records; this has been previously described [2,3].

Genome-wide genotyping was collected for a subset of 17,965 individuals, among which, 4049 men reported having been diagnosed with KC, with 1064 and 502 cases identified to have BCC and SCC based on pathological records, respectively. Healthy controls were selected from participants screened/self-reported to have no history of KC or actinic keratoses. Data on histology and location of the tumour were available for a subset of QSKIN participants (those identified to have BCC/SCC), which will be used in our MR analysis stratifying by body site of the cancer.

### Melanoma Institute of Australia

The Melanoma Institute of Australia is a non-profit organisation based in Poche Centre in North Sydney, Australia and affiliated with The University of Sydney. The MIA primarily conducts research and education programs on the preventive strategies and potential treatment for melanoma. The MIA melanoma study is one of the contributors to the GenoMEL Phase 2 melanoma GWAS meta-analysis. The MIA datasets were used for conducting body site-specific melanoma GWAS analyses to enable the stratified MR analysis by body site and by Breslow

thickness (see main text). We used these data conduct stratified GWAS analyses for male participants on **cutaneous** melanoma; all-body sites (2238 cases), head and neck (537 cases), trunk (1060 cases), arm (280 cases) and leg (361 cases); matching them against 979 healthy men as controls from the Australian Genetics of Depression Study (AGDS)[4]. We obtained male controls (979) from the Australian Genetics of Depression Study (AGDS), an Australian cohort of over 20,000 participants aged 43 years on average (SD=15 years) at the time of recruitment. Cohort details have been published elsewhere [4]. For the stratified analysis on Breslow thickness, we adopted the following criteria based on the AJCC 8th Edition T1 guidelines[5]. In brief, the MIA set was split into those whose first primary melanomas was thin (<=1 mm cut off used as is the max thickness) (n=765) and thick (>1 mm) (n=1,440).

In the present study, both MIA and AGDS samples were genotyped using the Illumina Global Screening Array V.2.0 (GSA) at the National Cancer Institute, USA. The MIA and AGDS studies were approved by the Human Research Ethics committee at QIMR Medical Research Institute in Brisbane and the Sydney Local Health District Ethics Review Committee at the Royal Prince Alfred Hospital in Sydney, Australia, respectively. All participants provided informed consent.

## Supplementary Notes 2. Definition of MPB in the UK Biobank

Data for male-pattern baldness is available for 227,354 men from the UK Biobank (UKB data-field 2395). In the UKB, MPB was defined using a 4 point scale, with each subsequent category/pattern indicating higher degree of baldness (see UKB resource 100423). We coded the MPB phenotype in the UKB as an ordinary phenotype, with larger values indicating increasing degree of baldness. Individuals reporting "do not know" were excluded in the analysis. We standardized the phenotype through an inverse-gaussian transformation prior to the GWAS analysis.

## Supplementary Notes 3. Derivation of free testosterone in the UK Biobank

Data on free/bioavailable testosterone is currently not available in the UK Biobank. However, we used the following second order equation proposed in Vermeulen et al.[6] to derive and estimate free testosterone through solving a mathematical equation involving total testosterone (field ID: 30850), SHBG (30830) and albumin concentration (30600).

$$S_{alb} = K_{alb} * C_{alb}/F_{alb}$$

$$SHBG_{bound} = SHBG_{total} - T_{total} + S_{alb} * T_{free}$$

yield $T_{free} = \frac{T_{total} - S_{alb} * T_{free}}{K_{SHBG} * SHBG_{bound}}$

Where $K_{alb}$ and $K_{SHBG}$ are defined at $K_{alb}$ =3.6*10^4 and $K_{SHBG}$ = 1*10^9. The factor $F_{alb}$ is set at 69000. $C_{alb}$ is the defined concentration of albumin (in g/L) while $T_{total}$ and $T_{free}$refers to the serum total and free testosterone concentration.

GWAS analysis was then conducted on the inverse-gaussian transformed free testosterone phenotype in white British males only using BOLT-LMM, having excluded individuals involved in the UKB KC GWAS.

## Supplementary Notes 4. Estimation of genetic association with MPB and endogenous sex-hormones from non-overlapping UKB subsample for use in MR analyses

To maximise power for instrument detection, we obtained candidate SNP instruments for our MR analyses for totalT, freeT and SHBG based on the GWAS findings on endogenous sex-hormones from Ruth et al. [15]. We obtained 473, 274 and 101 genome-wide significant variants as SNP instruments for SHBG, totalT and freeT. However, SNP effect sizes can be driven by winner's curse bias when genetic associations for the exposure and the outcome is estimated from the same sample. To avoid bias driven by sample overlap, we re-estimated the SNP effect size for each sex-hormones instrument obtained from Ruth et al. [15] in our UKB testosterone and SHBG GWAS derived from participants not involved in the outcome GWAS (i.e. KC GWASs). For simplicity, we reported all our MR estimates based on a 1 SD change in endogenous totalT, freeT or SHBG levels. A scatter plot showing the SNP effect size correlation between the Ruth et al [15]. GWAS and our UKB GWAS is shown in Supplementary Figures 2-4. Similarly, SNP instruments for MPB were identified from independent genome-wide significant variants in the unstratified GWAS on all UKB male participants for MPB, but we adopt only effect size estimates for each MPB SNPs from the non-overlapping set to facilitate the MR analyses.

## Supplementary Notes 5. Definition of site-specific skin cancers evaluated

To harness large sample sizes for our body-site specific skin cancer analysis, we collapsed . For ease of interpretation, we used the same category in both the melanoma and keratinocyte cancer analysis, where each of the body-site can be broadly classified as either [Head and neck], [Trunk], [Upper limb] or [Lower limb]. The number of cases in each category is shown in Supplementary Information - **Supplementary Table 2**.

## Supplementary Notes 6. Power calculation for MR analysis

The calculation of statistical power for the MR analysis was conducted through the online web interface, mRnd (available here https://cnsgenomics.com/shiny/mRnd/). To assess whether adequate power can be achieved for MR to detect effect sizes at various OR thresholds, nominal sample size for KC (as a benchmark) was provided along with the proportion of variance on exposure traits explained by SNP instruments. The proportion of variance explained by $m$ SNP instruments on the exposure of interest, $R^2$ can be derived through the formula below.

$$R^2 = \sum_{i=1}^{m} 2p_i(1 - p_i)\beta_i^2$$

Where $p_i$and $\beta_i$ refers to the effect allele frequency and the magnitude of association of the i-th SNP instrument with the exposure. Here we assume the exposure of interest (X) has been standardized prior to the analysis (i.e. var(X)~=1).

## Supplementary Notes 7. Estimates derived from alternative MR models

Here we adopted a series of alternative MR estimators [11] to triangulate findings from the inverse-variance weighted (IVW) model in the presence of weak violation on key MR assumptions, potential heterogeneity among SNP estimates and unmeasured pleiotropy. These estimators include: The MR-Egger, MR-Weighted Median, MR-PRESSO and the Weighted-mode model. Technical details behind the methodology of each model have been previously described [11]. Below is a short summary highlighting the key strength for each model to circumvent weaknesses of the IVW estimate:

*MR-Egger regression:* Provide estimates that are unbiased in the presence of directional pleiotropy, assuming the InSIDE (Instrument strength independent of direct effect) assumption is valid. The intercept for the MR-Egger regression provides indication for potential directional pleiotropy biasing IVW findings, when the intercept term is different from zero.
*MR-Mode based estimators:* Provide consistent estimates of MR effect sizes based on the plurality assumption.
*MR-Median based estimators:* Provide consistent estimates of MR effect sizes even when up to 50% of the SNP instruments are invalid.
*MR-PRESSO:* Identifies potential SNP outliers contributing to genetic heterogeneity, and provides adjusted estimates robust against horizontal pleiotropy and heterogeneity among SNP effect sizes. The MR-PRESSO outlier test was also used to detect potential SNP-outliers, of which putative candidate traits associated with the SNP-outlier can be incorporated in a multivariable MR framework.

## Supplementary Notes 8. Selection of pleiotropic variants for PheWAS analyses and validation via univariate MR analysis on MPB

The presence of horizontal pleiotropy between the SNP and outcome of interest can potentially invalidate the key MR assumptions. One way to identify SNP-confounder associations is to review the overall funnel plot/scatter plot for the MR association and evaluate potential outliers, or apply statistical models to test for outliers such as the outlier-test implemented in MR-PRESSO. However, these approaches very often remove potential causal effects implicated through a true vertical pleiotropic pathway (i.e. consistent with causality). To investigate whether the effect of identified pleiotropic variants are potentially linked with other putative skin-related risk factors in the same causal pathway on MPB, we performed the following. We applied MR-PRESSO [7] on the data (MPB against KC) to first obtain SNP-outliers driving genetic heterogeneity among the SNP-derived estimates. We used a total of 10,000 iterations to obtain stable distribution under the null in MR-PRESSO given that our SNP set is large (n~500 for MPB), and used a conservative SNP-outlier p-value cut-off of p<0.05. From there, we performed a PheWAS analysis on each outlier variant against a collection of pigmentation, and sun exposure phenotypes; as curated from the GeneATLAS[8] (see http://geneatlas.roslin.ed.ac.uk/) and OpenTarget[9] database (see https://genetics.opentargets.org/). Our inclusion criteria for candidate traits include any phenotype with the word “skin tones”, “pigmentation”, “sunburn”, “skin colour”, “hair colour”, “tanning”, “sunburn” and “skin related diseases”. For traits with multiple GWAS available, the association statistics with the strongest association P-value and largest sample size was preferentially reported.

For each meaningful SNP-trait pair association, we then performed a univariate MR analysis regressing MPB on the trait of interest (to check for directionality of the MR association). Trait-pairs with strong evidence (p<1e-5) for a MR association were then incorporated in a MVMR framework alongside testosterone and MPB against skin cancer outcomes. Description of the instrument selection criteria and data curation for the MVMR analysis is provided below (MVMR section). Our approach is essentially a modification and application of the concepts introduced by Cho et al. [10] in the MR-TRYX (Treasure your exceptions) framework to derive an adjusted marginal effect from the trait of interest (in our case, MPB) after accounting for alternative biological pathways driving the association between the SNP-outlier and the outcome (i.e. skin cancers).

Estimates derived from the IVW estimator will be preferentially reported in the main text. However, results derived using these sensitivity MR models can be found in Supplementary Tables (see Results section in main text).

## Supplementary Notes 9. Curation of instrument and assessment of instrument strength for MVMR analysis

MVMR analyses were performed to assess potential mediation effects to aid interpretation of our univariable MR analyses. Our SNP-outlier tests above revealed that both skin and hair colour are potentially associated with genetic MPB, and hence we incorporated both of these traits into our MVMR model. Note that we omitted the trait "ease of tanning" due to insufficient genetic predictors for MR. The GWASs for skin colour and hair colour conducted in the UK Biobank have been previously reported in another study (see Ong et al. [12]). We first combined all our SNP instruments (i.e. variants with genome-wide significant associations with the trait of interests) for each exposure of interest [hair colour, skin colour, totalT, freeT, SHBG and MPB] into one combined instrument set. We then removed duplicated signals by performing LD-clumping (kb=10000, r2=0.001) to ensure independence between SNP instruments, resulting in 1201 variants. Based on the newly curated set of 1201 SNPs, we then re-extract the effect size estimate for these SNPs on each exposure trait. SNPs with missing entries on at least one exposure trait or cannot be found in the outcome GWAS datasets were omitted from the analysis, resulting in 1132 variants. We assessed instrument strength for MVMR through the strength_mvmr() function implemented via the *MendelianRandomization* R package [13]. The MVMR analyses were performed using the mv_multiple() function in the *TwoSampleMR* R package. In our main analysis we provided estimates for two selected models **(**Supplementary Information - **Supplementary Table 5)**.

*Original MVMR model (without modeling heterogeneous outlier).* Initially, the conditional F-statistics based on our curated instrument set for the original 4 trait model [MPB, totalT, freeT, SHBG] is 28.87, 0.21 0.21 and 0.22 (See **Supplementary Table 5**). We then repeated the instrument curation process by excluding SHBG from our analyses due to the SHBG phenotype having the lowest correlation with both MPB and testosterone phenotypes and calculated the conditional F-statistics again. The conditional F-statistics in the reduced set (n=262 SNPs) is 10.5, 55.6 and 16.1 for freeT, MPB and totalT respectively, indicating that our curated instrument set satisfies the strong instrument criteria (cond.F>10) for MVMR [14]. We hence omitted the SHBG from the MVMR analyses for the original model. MVMR estimates derived from this model were also provided in Supplementary Information - **Supplementary Table 10**.

*MVMR model with proxy traits tagging heterogenous SNP outliers.* For the MVMR model involving the two other proxy traits (obtained via modeling PheWAS associations on genetic outliers; see Supplementary Notes section *Selection of pleiotropic variants for PheWAS analyses and validation via univariate MR analysis on MPB*) namely hair colour and skin colour, we selected model D1 on the basis of instrument strength for MVMR by excluding SHBG and freeT from the analysis (4 trait model yield better instrumentstrength than 6 trait model; See Supplementary Information - **Supplementary Table 5**).

# Supplementary Tables

## Supplementary Table 1. Description of cases and controls for individual studies involved in the GenoMEL Phase 2 GWAS meta-analysis.

| Population | Study | Cases | Controls | Cases (males only) | Controls (males only) | Effective cases | Effective controls |
|---|---|---|---|---|---|---|---|
| European | GenoMEL Phase 1 | 1,075 | 2,163 | 449 | 893 | 1,436 | 1,436 |
| European | GenoMEL Phase 2 | 925 | 1,128 | 356 | 520 | 1,016 | 1,016 |
| US | MDACC | 1,924 | 1,018 | 1,129 | 608 | 1,331 | 1,331 |
| Australian | AMFS | 535 | 427 | 176 | 177 | 475 | 475 |
| Australian | Q-MEGA_610k | 912 | 3,777 | 421 | 760 | 1,469 | 1,469 |
| Australian | Q-MEGA_omni | 656 | 538 | 316 | 422 | 591 | 591 |
| UK | GSEdinCIDRulcer | 4,328 | 5,780 | 1,879 | 2,435 | 4,950 | 4,950 |
| Australian | WAMHS | 1,237 | 1,977 | 720 | 932 | 1,523 | 1,523 |
| German | Essen-Heidelberg | 1,189 | 1,215 | 664 | 607 | 1,201 | 1,201 |
| French | MELARISK | 511 | 815 | | | 628 | 628 |
| US | Harvard | 410 | 2,920 | | | 719 | 719 |
| US | NCI_CPSII+PLCO+Rose | 171 | 2,684 | | | 322 | 322 |
| UK | UK Biobank confirmed | 3,499 | 13,996 | 1,438 | 6,344 | 5,598 | 5,598 |
| European-derived | MIA_PAH | 1,933 | 2,841 | | | 2,301 | 2,301 |
| Australian | EPIGENE | 773 | 910 | 510 | 392 | 835 | 835 |
| Australian | QSKIN | 1,285 | 2,493 | | | 1696 | 1696 |
| Greek | Greek | 1,194 | 1,279 | 605 | 578 | 1235 | 1235 |
| Italian | Italy | 1,726 | 3,142 | 786 | 2169 | 2228 | 2228 |
| Spanish | Spain | 3,523 | 3,400 | 1604 | 2105 | 3460 | 3460 |
| US | Michigan | 1,198 | 26,211 | | | 2,291 | 2,291 |
| European-derived | BNMS | 1,130 | 2,701 | | | 1593 | 1593 |
| | **Total confirmed** | **30,134** | **81,415** | 11,053 | 18,943 | **36,898** | **36,898** |
| UK | UK Biobank SR | 1,802 | 7,208 | 843 | 3,319 | 2,888 | 2,888 |
| International | 23andMe SR | 4,824 | 286,565 | | | 9,488 | 9,488 |
| | **Total all Samples** | **36,760** | **375,188** | 11,896 | 22,262 | **49,274** | **49,274** |

Cf=confirmed. SR=self-reported

## Supplementary Table 2. Distribution of cases and controls for analyses on major body-site categories

| Trait | Site | Cohort | Cases | Controls |
|---|---|---|---|---|
| *Distribution across all sites* | | | | |
| KC | all | UKB | 13,463 | 96,620 |
| | all | QSkin | 4,049 | 1,957 |
| BCC | all | UKB | 10,718 | 96,620 |
| | all | QSkin | 992 | 1,957 |
| SCC | all | UKB | 3,483 | 96,620 |
| | all | QSkin | 462 | 1,957 |
| *Distribution across individual body-site categories* | | | | |
| BCC | Head and Neck | QSkin | 439 | 1,957 |
| | Trunk | QSkin | 209 | 1,957 |
| | Upper limb | QSkin | 248 | 1,957 |
| | Lower limb | QSkin | 93 | 1,957 |
| SCC | Head and Neck | QSkin | 184 | 1,957 |
| | Trunk | QSkin | 23 | 1,957 |
| | Upper limb | QSkin | 149 | 1,957 |
| | Lower limb | QSkin | 102 | 1,957 |
| CM | all | MIA | 2,236 | 3,176 |
| | all (thin: <= 1mm Breslow thickness) | MIA | 765 | 3,176 |
| | all (thick: > 1mm Breslow thickness) | MIA | 1,440 | 3,176 |
| | Head and Neck* | MIA | 537 | 3,176 |
| | Head and Neck (no scalp) | MIA | 364 | 3,176 |
| | Trunk | MIA | 883 | 3,176 |
| | Upper limb | MIA | 448 | 3,176 |
| | Lower limb | MIA | 368 | 3,176 |
| | Scalp | MIA | 173 | 3,176 |
| KC | Head and Neck** | UKB | 6,114 | 96,620 |
| | Trunk | UKB | 1,709 | 96,620 |
| | Upper limb | UKB | 922 | 96,620 |
| | Lower limb | UKB | 449 | 96,620 |
| | Scalp and neck | UKB | 996 | 96,620 |
| BCC | Head and Neck** | UKB | 4,768 | 96,620 |
| | Trunk | UKB | 1,442 | 96,620 |
| | Upper limb | UKB | 636 | 96,620 |
| | Lower limb | UKB | 301 | 96,620 |

| | | | | |
|---|---|---|---|---|
| | Scalp and neck | UKB | 650 | 96,620 |
| SCC | Head and Neck** | UKB | 1,427 | 96,620 |
| | Trunk | UKB | 298 | 96,620 |
| | Upper limb | UKB | 284 | 96,620 |
| | Lower limb | UKB | 143 | 96,620 |
| | Scalp and neck | UKB | 325 | 96,620 |
| CM | Head and Neck** | UKB | 390 | 96,620 |
| | Trunk | UKB | 925 | 96,620 |
| | Upper limb | UKB | 446 | 96,620 |
| | Lower limb | UKB | 290 | 96,620 |
| | Scalp and neck | UKB | 137 | 96,620 |

*includes scalp

**includes scalp and neck

## Supplementary Table 3. Body-site specific sample size distribution for melanoma and keratinocyte cancer cases in the UK Biobank

| Body region | Specific site definition | CM codes (ICD10/9) | KC codes (ICD10/9) | CM | KC - BCC | KC - SCC |
|---|---|---|---|---|---|---|
| **Head and Neck** | Lip | C43.0/1720 | C44.0/1730 | 1 | 80 | 48 |
| | Eyelid, including canthus | C43.1/1721 | C44.1/1731 | 9 | 409 | 66 |
| | Ear and external auricular canal | C43.2/1722 | C44.2/1732 | 71 | 504 | 257 |
| | Other and unspecified parts of face | C43.3/1723 | C44.3/1733 | 172 | 3125 | 731 |
| | Scalp and neck | C43.4/1724 | C44.4/1734 | 137 | 650 | 325 |
| | | | | | | |
| **Trunk** | Trunk | C43.5/1725 | C44.5/1735 | 925 | 1442 | 298 |
| | | | | | | |
| **Upper limb** | Upper limb, including shoulder | C43.6/1726 | C44.6/1736 | 446 | 636 | 284 |
| | | | | | | |
| **Lower limb** | Lower limb, including hip | C43.7/1727 | C44.7/1737 | 290 | 301 | 143 |

ICD10=International Classification of Diseases version 10. ICD9=International Classification of Diseases version 9. Body region are defined as anatomical categories covering specific body sites, for our analysis.

## Supplementary Table 4. Body-site specific sample size distribution for melanoma and keratinocyte cancer cases in the Australian (QSKIN and MIA) datasets

| Body region | Specific site | Diagnosis | | |
|---|---|---|---|---|
| | | CM (MIA) | BCC (QSKIN) | SCC (QSKIN) |
| **Head and neck** | Central forehead | 1 | - | - |
| | Ear | 59 | 57 | 28 |
| | Cheek | 91 | - | - |
| | Chin | 4 | - | - |
| | Face | 16 | 299 | 112 |
| | Scalp | 173 | 24 | 21 |
| | Nose | 19 | - | - |
| | Neck | 100 | 59 | 23 |
| | Jaw | 1 | - | - |
| | Eyelid | 3 | - | - |
| | Lip | 5 | - | - |
| | Forehead & temple | 64 | - | - |
| | Conjuctiva | 1 | - | - |
| | | | | |
| **Trunk** | Lower abdomen | 6 | - | - |
| | Abdomen (NOS) | 28 | 8 | 2 |
| | Upper abdomen\upper chest | 18 | 54 | 10 |
| | Anus | 1 | - | - |
| | Back | 3 | 34 | 2 |
| | Caudal back | 1 | - | - |
| | Central back | 1 | - | - |
| | Interscapular back | 1 | - | - |
| | Left back | 1 | - | - |
| | Lower back | 40 | 22 | 2 |
| | Mid back | 3 | - | - |
| | Mid right back | 5 | - | - |
| | Central sternum | 1 | - | - |
| | Anterior thorax | 118 | 17 | 3 |
| | Posterior thorax (upper back) | 594 | 74 | 4 |
| | Thorax (NOS) | 18 | - | - |

| | | | | |
|---|---|---|---|---|
| | Left chest wall | 1 | - | - |
| | Right Paravertebral (upper back) | 1 | - | - |
| | Flank | 38 | - | - |
| | Left flank | 1 | - | - |
| | Groin | 1 | - | - |
| | Left loin | 1 | - | - |
| | Penis | 1 | - | - |
| | | | | |
| **Upper Limb** | Shoulder | 164 | 103 | 13 |
| | Arm | 134 | 55 | 19 |
| | Forearm | 121 | 80 | 63 |
| | Tricep | 1 | - | - |
| | Elbow | 23 | - | - |
| | Back of hand | - | 9 | 48 |
| | Wrist | 1 | - | - |
| | Clavicular region | 1 | - | - |
| | Scapula | 2 | - | - |
| | Palmer skin | - | 1 | 6 |
| | Suprascapular region | 1 | - | - |
| | | | | |
| **Lower Limb** | Thigh | 98 | 9 | 8 |
| | Knee | 30 | - | - |
| | Calf | 132 | - | - |
| | Lower leg | 2 | - | - |
| | Shin | 80 | - | - |
| | Leg (NOS) | 3 | 79 | 86 |
| | Ankle | 16 | - | - |
| | Buttock | 7 | - | - |
| | Top of foot | - | 4 | 7 |
| | Hip | - | 1 | 1 |

Note that all reported diagnosis are clinically confirmed diagnosis (not self-reports). We excluded specific sites that have no diagnosis instances across the 3 disease (CM, BCC, SCC) evaluated.

## Supplementary Table 5. Conditional F-statistics to measure instrument strength for multivariable MR in various combination of traits

| Traits | Model O (359 SNPs) | | **Model A (262 SNPs)** | | Model B (538 SNPs) | | Model C (578 SNPs) | | Model D (556 SNPs) | | **Model D1 (560 SNPs)** | | Model E (1132 SNPs) | |
|---|---|---|---|---|---|---|---|---|---|---|---|---|---|---|
| | Inc. | F | Inc. | F | Inc. | F | Inc. | F | Inc. | F | Inc. | F | Inc. | F |
| MPB | T | 28.87 | T | 55.6 | T | 25.01 | T | 22.97 | T | 23.7 | T | 23.54 | T | 12.1 |
| totalT | T | 0.21 | T | 16.1 | T | 8.49 | T | 7.98 | T | 15.66 | T | 15.72 | T | 0.19 |
| freeT | T | 0.21 | T | 10.5 | T | 5.37 | T | 5.04 | F | - | F | - | T | 0.19 |
| SHBG | T | 0.22 | F | - | F | | F | - | F | - | F | - | T | 0.2 |
| skin colour | - | | - | - | F | | T | 31.01 | T | 31.85 | T | 8.6 | T | 16.85 |
| hair colour | - | | - | - | T | 130.85 | T | 75.38 | T | 77.44 | T | 14.04 | T | 33.04 |

Inc.=Include trait. T=True; F=False.

for MVMR: to obtain these subsets, we reclump the instruments, by retaining only genome-wide variants (p<5e-8) with the trait of interest as genetic instruments. for this reason, the MVMR SNP instrument for each individual trait might be more robust than its univariable counterpart (as we re-define instrument as SNPs that have association p<5e-8 in the UK Biobank non-overlapping subset itself).

## Supplementary Table 6. Estimated univariate MR derived ORs for per 1 SD change increase in genetically predicted MPB score on skin cancer risk

| outcome | exposure | method | All variants | | | Variants with p<1e-5 in subset | | |
|---|---|---|---|---|---|---|---|---|
| | | | nsnp | pval | OR | nsnp | pval | OR |
| BCC QSKIN | MPB | MR Egger | 471 | 0.06 | 1.6 (0.98 to 2.61) | 287 | 0.1 | 1.70 (0.91 to 3.15) |
| BCC QSKIN | MPB | Weighted median | 471 | 0.86 | 0.97 (0.66 to 1.41) | 287 | 0.97 | 0.99 (0.67 to 1.46) |
| BCC QSKIN | MPB | IVW (fixed effect) | 471 | 0.49 | 1.1 (0.84 to 1.43) | 287 | 0.31 | 1.17 (0.87 to 1.58) |
| BCC QSKIN | MPB | Simple mode | 471 | 0.09 | 0.45 (0.18 to 1.12) | 287 | 0.32 | 0.61 (0.23 to 1.61) |
| BCC QSKIN | MPB | Weighted mode | 471 | 0.37 | 0.79 (0.48 to 1.32) | 287 | 0.43 | 0.80 (0.45 to 1.41) |
| BCC UKB | MPB | MR Egger | 472 | 7.76E-03 | 1.26 (1.06 to 1.49) | 287 | 0.01 | 1.37 (1.09 to 1.72) |
| BCC UKB | MPB | Weighted median | 472 | 0.36 | 1.05 (0.95 to 1.16) | 287 | 0.38 | 1.05 (0.94 to 1.16) |
| BCC UKB | MPB | IVW (fixed effect) | 472 | 1.84E-03 | 1.15 (1.05 to 1.26) | 287 | 0.02 | 1.14 (1.02 to 1.28) |
| BCC UKB | MPB | Simple mode | 472 | 0.46 | 1.1 (0.86 to 1.41) | 287 | 0.72 | 1.05 (0.81 to 1.37) |
| BCC UKB | MPB | Weighted mode | 472 | 0.99 | 1 (0.87 to 1.15) | 287 | 0.85 | 0.99 (0.85 to 1.14) |
| KC QSKIN | MPB | MR Egger | 470 | 0.03 | 1.49 (1.05 to 2.11) | 286 | 0.14 | 1.41 (0.90 to 2.20) |
| KC QSKIN | MPB | Weighted median | 470 | 0.66 | 0.94 (0.73 to 1.23) | 286 | 0.72 | 0.95 (0.72 to 1.25) |
| KC QSKIN | MPB | IVW (fixed effect) | 470 | 0.78 | 1.03 (0.85 to 1.24) | 286 | 0.35 | 1.11 (0.89 to 1.37) |
| KC QSKIN | MPB | Simple mode | 470 | 0.46 | 0.79 (0.42 to 1.48) | 286 | 0.76 | 0.90 (0.46 to 1.77) |
| KC QSKIN | MPB | Weighted mode | 470 | 0.98 | 1.01 (0.69 to 1.47) | 286 | 0.97 | 1.01 (0.67 to 1.51) |
| KC UKB | MPB | MR Egger | 472 | 7.99E-03 | 1.23 (1.06 to 1.43) | 287 | 0.01 | 1.32 (1.07 to 1.63) |
| KC UKB | MPB | Weighted median | 472 | 0.28 | 1.05 (0.96 to 1.15) | 287 | 0.31 | 1.05 (0.96 to 1.16) |
| KC UKB | MPB | IVW (fixed effect) | 472 | 1.45E-04 | 1.17 (1.08 to 1.27) | 287 | 3.86E-03 | 1.16 (1.05 to 1.28) |
| KC UKB | MPB | Simple mode | 472 | 0.72 | 1.04 (0.83 to 1.3) | 287 | 0.9 | 1.02 (0.80 to 1.29) |
| KC UKB | MPB | Weighted mode | 472 | 0.83 | 0.99 (0.87 to 1.11) | 287 | 0.86 | 0.99 (0.87 to 1.12) |
| Melanoma (phase2) | MPB | MR Egger | 469 | 0.9 | 0.99 (0.85 to 1.15) | 286 | 0.97 | 1.00 (0.83 to 1.19) |
| Melanoma (phase2) | MPB | Weighted median | 469 | 0.64 | 0.97 (0.87 to 1.09) | 286 | 0.6 | 0.97 (0.86 to 1.09) |
| Melanoma (phase2) | MPB | IVW (fixed effect) | 469 | 0.76 | 0.99 (0.91 to 1.07) | 286 | 0.38 | 0.96 (0.88 to 1.05) |
| Melanoma (phase2) | MPB | Simple mode | 469 | 0.06 | 1.32 (0.99 to 1.76) | 286 | 0.28 | 1.19 (0.87 to 1.62) |
| Melanoma (phase2) | MPB | Weighted mode | 469 | 0.3 | 1.09 (0.93 to 1.27) | 286 | 0.29 | 1.10 (0.92 to 1.31) |
| SCC QSKIN | MPB | MR Egger | 471 | 0.05 | 1.93 (1.01 to 3.71) | 287 | 0.04 | 2.41 (1.06 to 5.50) |

| | | | | | | | | |
|---|---|---|---|---|---|---|---|---|
| SCC QSKIN | MPB | Weighted median | 471 | 0.78 | 1.07 (0.65 to 1.76) | 287 | 0.7 | 1.10 (0.66 to 1.84) |
| SCC QSKIN | MPB | IVW (fixed effect) | 471 | 0.65 | 1.08 (0.77 to 1.53) | 287 | 0.48 | 1.15 (0.77 to 1.72) |
| SCC QSKIN | MPB | Simple mode | 471 | 0.66 | 0.76 (0.22 to 2.61) | 287 | 0.52 | 0.63 (0.15 to 2.57) |
| SCC QSKIN | MPB | Weighted mode | 471 | 0.73 | 1.14 (0.55 to 2.37) | 287 | 0.68 | 1.19 (0.52 to 2.77) |
| SCC UKB | MPB | MR Egger | 472 | 0.05 | 1.24 (1 to 1.53) | 287 | 0.09 | 1.27 (0.97 to 1.67) |
| SCC UKB | MPB | Weighted median | 472 | 7.45E-03 | 1.24 (1.06 to 1.46) | 287 | 0.01 | 1.24 (1.06 to 1.46) |
| SCC UKB | MPB | IVW (fixed effect) | 472 | 2.35E-06 | 1.31 (1.17 to 1.46) | 287 | 1.33E-04 | 1.29 (1.13 to 1.48) |
| SCC UKB | MPB | Simple mode | 472 | 0.16 | 1.31 (0.9 to 1.89) | 287 | 0.11 | 1.37 (0.93 to 2.01) |
| SCC UKB | MPB | Weighted mode | 472 | 0.04 | 1.23 (1.01 to 1.49) | 287 | 0.12 | 1.21 (0.95 to 1.54) |

Note: The fixed-effect meta-analysed *ORs combining QSKIN and UKB estimates can be found in the main text.*
Estimates for melanoma (phase 2) was derived from the GenoMEL Phase 2 melanoma GWAS meta-analysis which includes the cases from the MIA datasets.

## Supplementary Table 7. Estimated univariate MR derived ORs for per 1 SD change increase in genetically predicted free testosterone on skin cancer risk

| outcome | exposure | method | All variants | | | Variants with p<1e-5 in subset | | |
|---|---|---|---|---|---|---|---|---|
| | | | **nsnp** | **pval** | OR | **nsnp** | **pval** | **OR** |
| BCC QSKIN | freeT | MR Egger | 74 | 0.73 | 0.86 (0.36 to 2.07) | 56 | 0.87 | 0.93 (0.39 to 2.21) |
| BCC QSKIN | freeT | Weighted median | 74 | 0.73 | 0.89 (0.44 to 1.78) | 56 | 0.75 | 0.89 (0.43 to 1.84) |
| BCC QSKIN | freeT | IVW (fixed effect) | 74 | 0.75 | 0.93 (0.57 to 1.5) | 56 | 0.54 | 0.87 (0.55 to 1.37) |
| BCC QSKIN | freeT | Simple mode | 74 | 0.67 | 0.76 (0.21 to 2.73) | 56 | 0.91 | 0.93 (0.27 to 3.16) |
| BCC QSKIN | freeT | Weighted mode | 74 | 0.49 | 0.76 (0.35 to 1.65) | 56 | 0.39 | 0.73 (0.35 to 1.50) |
| BCC UKB | freeT | MR Egger | 86 | 0.56 | 0.91 (0.68 to 1.24) | 66 | 0.67 | 0.93 (0.66 to 1.31) |
| BCC UKB | freeT | Weighted median | 86 | 0.66 | 0.95 (0.78 to 1.17) | 66 | 0.6 | 0.95 (0.77 to 1.16) |
| BCC UKB | freeT | IVW (fixed effect) | 86 | 0.85 | 0.98 (0.83 to 1.16) | 66 | 0.77 | 0.97 (0.81 to 1.17) |
| BCC UKB | freeT | Simple mode | 86 | 0.06 | 1.52 (1 to 2.32) | 66 | 0.21 | 1.32 (0.86 to 2.03) |
| BCC UKB | freeT | Weighted mode | 86 | 0.42 | 0.91 (0.72 to 1.15) | 66 | 0.42 | 0.91 (0.72 to 1.15) |
| KC QSKIN | freeT | MR Egger | 74 | 0.66 | 1.18 (0.58 to 2.39) | 56 | 0.56 | 1.25 (0.60 to 2.60) |
| KC QSKIN | freeT | Weighted median | 74 | 0.72 | 0.91 (0.55 to 1.51) | 56 | 0.74 | 0.91 (0.53 to 1.57) |
| KC QSKIN | freeT | IVW (fixed effect) | 74 | 0.89 | 0.97 (0.66 to 1.44) | 56 | 0.74 | 0.94 (0.63 to 1.39) |
| KC QSKIN | freeT | Simple mode | 74 | 0.76 | 1.15 (0.47 to 2.79) | 56 | 0.79 | 1.13 (0.47 to 2.68) |
| KC QSKIN | freeT | Weighted mode | 74 | 0.92 | 0.97 (0.55 to 1.72) | 56 | 0.75 | 0.91 (0.51 to 1.62) |
| KC UKB | freeT | MR Egger | 86 | 0.47 | 0.9 (0.69 to 1.19) | 66 | 0.58 | 0.92 (0.67 to 1.25) |
| KC UKB | freeT | Weighted median | 86 | 0.27 | 0.91 (0.77 to 1.08) | 66 | 0.29 | 0.91 (0.76 to 1.08) |
| KC UKB | freeT | IVW (fixed effect) | 86 | 0.61 | 0.96 (0.83 to 1.12) | 66 | 0.61 | 0.96 (0.81 to 1.13) |
| KC UKB | freeT | Simple mode | 86 | 0.45 | 1.17 (0.78 to 1.73) | 66 | 0.51 | 1.14 (0.78 to 1.66) |
| KC UKB | freeT | Weighted mode | 86 | 0.25 | 0.9 (0.75 to 1.08) | 66 | 0.27 | 0.89 (0.73 to 1.09) |
| melanoma | freeT | MR Egger | 74 | 0.24 | 0.8 (0.55 to 1.16) | 56 | 0.27 | 0.78 (0.51 to 1.21) |
| melanoma | freeT | Weighted median | 74 | 0.31 | 0.89 (0.7 to 1.12) | 56 | 0.28 | 0.88 (0.70 to 1.11) |
| melanoma | freeT | IVW (fixed effect) | 74 | 0.91 | 0.99 (0.81 to 1.2) | 56 | 0.84 | 0.98 (0.78 to 1.22) |

| | | | | | | | | |
|---|---|---|---|---|---|---|---|---|
| melanoma | freeT | Simple mode | 74 | 0.93 | 1.02 (0.66 to 1.57) | 56 | 0.88 | 1.03 (0.67 to 1.61) |
| melanoma | freeT | Weighted mode | 74 | 0.3 | 0.88 (0.7 to 1.11) | 56 | 0.33 | 0.88 (0.68 to 1.14) |
| SCC QSKIN | freeT | MR Egger | 74 | 0.31 | 1.78 (0.58 to 5.39) | 56 | 0.34 | 1.80 (0.54 to 5.94) |
| SCC QSKIN | freeT | Weighted median | 74 | 0.63 | 1.26 (0.49 to 3.24) | 56 | 0.65 | 1.26 (0.46 to 3.44) |
| SCC QSKIN | freeT | IVW (fixed effect) | 74 | 0.82 | 1.07 (0.58 to 1.99) | 56 | 0.79 | 1.09 (0.57 to 2.09) |
| SCC QSKIN | freeT | Simple mode | 74 | 0.82 | 1.22 (0.21 to 7) | 56 | 0.78 | 1.28 (0.23 to 7.13) |
| SCC QSKIN | freeT | Weighted mode | 74 | 0.9 | 1.08 (0.34 to 3.41) | 56 | 0.94 | 1.04 (0.35 to 3.13) |
| SCC UKB | freeT | MR Egger | 86 | 0.77 | 0.94 (0.64 to 1.39) | 66 | 0.8 | 0.95 (0.64 to 1.41) |
| SCC UKB | freeT | Weighted median | 86 | 0.36 | 0.88 (0.66 to 1.16) | 66 | 0.37 | 0.88 (0.67 to 1.16) |
| SCC UKB | freeT | IVW (fixed effect) | 86 | 0.46 | 0.92 (0.75 to 1.14) | 66 | 0.59 | 0.94 (0.77 to 1.16) |

**Supplementary Table 8. Estimated univariate MR derived ORs for per 1 SD change increase in genetically predicted total testosterone on skin cancer risk**

| outcome | exposure | method | All variants | | | Variants with p<1e-5 in subset | | |
|---|---|---|---|---|---|---|---|---|
| | | | nsnp | pval | OR | nsnp | pval | OR |
| BCC QSKIN | totalT | MR Egger | 152 | 0.66 | 0.9 (0.54 to 1.47) | 99 | 0.35 | 0.78 (0.46 to 1.32) |
| BCC QSKIN | totalT | Weighted median | 15 | 0.3 | 0.76 (0.44 to 1.28) | 99 | 0.28 | 0.75 (0.45 to 1.26) |
| BCC QSKIN | totalT | IVW (fixed effect) | 152 | 0.29 | 1.18 (0.86 to 1.62) | 99 | 0.5 | 1.12 (0.81 to 1.55) |
| BCC QSKIN | totalT | Simple mode | 152 | 0.82 | 1.15 (0.35 to 3.81) | 99 | 0.66 | 1.33 (0.37 to 4.84) |
| BCC QSKIN | totalT | Weighted mode | 152 | 0.39 | 0.79 (0.47 to 1.34) | 99 | 0.38 | 0.77 (0.42 to 1.38) |
| BCC UKB | totalT | MR Egger | 168 | 0.6 | 0.96 (0.83 to 1.12) | 111 | 0.58 | 0.95 (0.81 to 1.13) |
| BCC UKB | totalT | Weighted median | 168 | 0.57 | 1.05 (0.9 to 1.22) | 111 | 0.6 | 1.04 (0.90 to 1.21) |
| BCC UKB | totalT | IVW (fixed effect) | 168 | 0.37 | 1.04 (0.95 to 1.15) | 111 | 0.45 | 1.04 (0.94 to 1.15) |
| BCC UKB | totalT | Simple mode | 168 | 0.84 | 0.97 (0.71 to 1.32) | 111 | 0.85 | 0.97 (0.71 to 1.33) |
| BCC UKB | totalT | Weighted mode | 168 | 0.97 | 1 (0.88 to 1.15) | 111 | 0.81 | 1.02 (0.88 to 1.17) |
| KC QSKIN | totalT | MR Egger | 152 | 0.95 | 1.01 (0.67 to 1.54) | 99 | 0.52 | 0.86 (0.55 to 1.35) |
| KC QSKIN | totalT | Weighted median | 152 | 0.22 | 0.79 (0.54 to 1.15) | 99 | 0.23 | 0.79 (0.53 to 1.17) |
| KC QSKIN | totalT | IVW (fixed effect) | 152 | 0.3 | 1.15 (0.88 to 1.5) | 99 | 0.32 | 1.15 (0.87 to 1.52) |
| KC QSKIN | totalT | Simple mode | 152 | 0.7 | 1.21 (0.46 to 3.13) | 99 | 0.82 | 1.11 (0.45 to 2.78) |
| KC QSKIN | totalT | Weighted mode | 152 | 0.41 | 0.86 (0.6 to 1.23) | 99 | 0.37 | 0.84 (0.58 to 1.23) |
| KC UKB | totalT | MR Egger | 168 | 0.63 | 0.97 (0.85 to 1.1) | 111 | 0.58 | 0.96 (0.83 to 1.11) |
| KC UKB | totalT | Weighted median | 168 | 0.54 | 1.04 (0.91 to 1.19) | 111 | 0.55 | 1.04 (0.91 to 1.20) |
| KC UKB | totalT | IVW (fixed effect) | 168 | 0.83 | 1.01 (0.93 to 1.1) | 111 | 0.81 | 1.01 (0.93 to 1.10) |
| KC UKB | totalT | Simple mode | 168 | 0.8 | 1.04 (0.78 to 1.39) | 111 | 1 | 1.00 (0.76 to 1.32) |
| KC UKB | totalT | Weighted mode | 168 | 0.98 | 1 (0.88 to 1.13) | 111 | 0.99 | 1.00 (0.87 to 1.15) |
| melanoma | totalT | MR Egger | 151 | 0.28 | 0.91 (0.76 to 1.08) | 99 | 0.23 | 0.89 (0.73 to 1.08) |

| | | | | | | | | |
|---|---|---|---|---|---|---|---|---|
| melanoma | totalT | Weighted median | 151 | 0.65 | 0.96 (0.8 to 1.15) | 99 | 0.66 | 0.96 (0.80 to 1.16) |
| melanoma | totalT | IVW (fixed effect) | 151 | 0.29 | 0.94 (0.84 to 1.05) | 99 | 0.34 | 0.94 (0.84 to 1.06) |
| melanoma | totalT | Simple mode | 151 | 0.69 | 1.07 (0.76 to 1.5) | 99 | 0.56 | 1.11 (0.79 to 1.56) |
| melanoma | totalT | Weighted mode | 151 | 0.54 | 0.95 (0.81 to 1.12) | 99 | 0.57 | 0.95 (0.81 to 1.12) |
| SCC QSKIN | totalT | MR Egger | 152 | 0.86 | 1.06 (0.55 to 2.05) | 99 | 0.65 | 0.85 (0.42 to 1.72) |
| SCC QSKIN | totalT | Weighted median | 15 | 0.21 | 0.66 (0.35 to 1.26) | 99 | 0.2 | 0.64 (0.33 to 1.27) |
| SCC QSKIN | totalT | IVW (fixed effect) | 152 | 0.31 | 1.24 (0.82 to 1.87) | 99 | 0.42 | 1.19 (0.77 to 1.84) |
| SCC QSKIN | totalT | Simple mode | 152 | 0.57 | 1.5 (0.36 to 6.17) | 99 | 0.79 | 1.21 (0.30 to 4.99) |
| SCC QSKIN | totalT | Weighted mode | 152 | 0.61 | 0.85 (0.45 to 1.59) | 99 | 0.46 | 0.77 (0.39 to 1.52) |
| SCC UKB | totalT | MR Egger | 168 | 0.93 | 0.99 (0.8 to 1.23) | 111 | 0.75 | 0.96 (0.77 to 1.21) |
| SCC UKB | totalT | Weighted median | 168 | 0.73 | 0.96 (0.76 to 1.22) | 111 | 0.74 | 0.96 (0.75 to 1.23) |
| SCC UKB | totalT | IVW (fixed effect) | 168 | 0.24 | 0.92 (0.81 to 1.05) | 111 | 0.49 | 0.95 (0.83 to 1.09) |
| SCC UKB | totalT | Simple mode | 168 | 0.85 | 1.05 (0.65 to 1.69) | 111 | 0.71 | 1.09 (0.68 to 1.76) |
| SCC UKB | totalT | Weighted mode | 168 | 0.79 | 0.97 (0.79 to 1.19) | 111 | 0.73 | 0.96 (0.76 to 1.21) |

## Supplementary Table 9. Estimated univariate MR derived ORs for per 1 SD change increase in genetically predicted SHBG on skin cancer risk

| outcome | exposure | method | All variants | | | Variants with p<1e-5 in subset | | |
|---|---|---|---|---|---|---|---|---|
| | | | nsnp | pval | OR | nsnp | pval | OR |
| BCC QSKIN | SHBG | MR Egger | 234 | 0.42 | 1.15 (0.82 to 1.61) | 226 | 0.47 | 1.13 (0.81 to 1.59) |
| BCC QSKIN | SHBG | Weighted median | 234 | 0.62 | 0.91 (0.63 to 1.32) | 226 | 0.63 | 0.92 (0.64 to 1.31) |
| BCC QSKIN | SHBG | IVW (fixed effect) | 234 | 0.39 | 1.11 (0.88 to 1.39) | 226 | 0.35 | 1.11 (0.89 to 1.40) |
| BCC QSKIN | SHBG | Simple mode | 234 | 0.48 | 1.37 (0.58 to 3.24) | 226 | 0.46 | 1.36 (0.60 to 3.11) |
| BCC QSKIN | SHBG | Weighted mode | 234 | 0.52 | 1.12 (0.8 to 1.55) | 226 | 0.54 | 1.11 (0.79 to 1.56) |
| BCC UKB | SHBG | MR Egger | 262 | 0.79 | 0.98 (0.87 to 1.11) | 254 | 0.74 | 0.98 (0.87 to 1.11) |
| BCC UKB | SHBG | Weighted median | 262 | 0.54 | 1.03 (0.93 to 1.15) | 254 | 0.54 | 1.03 (0.93 to 1.15) |
| BCC UKB | SHBG | IVW (fixed effect) | 262 | 0.13 | 1.07 (0.98 to 1.16) | 254 | 0.12 | 1.07 (0.98 to 1.16) |
| BCC UKB | SHBG | Simple mode | 262 | 0.81 | 1.03 (0.83 to 1.27) | 254 | 0.82 | 1.03 (0.82 to 1.29) |
| BCC UKB | SHBG | Weighted mode | 262 | 0.53 | 1.03 (0.95 to 1.11) | 254 | 0.53 | 1.03 (0.95 to 1.11) |
| KC QSKIN | SHBG | MR Egger | 233 | 0.86 | 0.98 (0.76 to 1.26) | 225 | 0.85 | 0.97 (0.76 to 1.26) |
| KC QSKIN | SHBG | Weighted median | 233 | 0.27 | 0.86 (0.65 to 1.13) | 225 | 0.26 | 0.86 (0.65 to 1.12) |
| KC QSKIN | SHBG | IVW (fixed effect) | 233 | 0.59 | 1.05 (0.88 to 1.24) | 225 | 0.59 | 1.05 (0.88 to 1.25) |
| KC QSKIN | SHBG | Simple mode | 233 | 0.01 | 0.38 (0.18 to 0.81) | 225 | 0.01 | 0.39 (0.18 to 0.83) |
| KC QSKIN | SHBG | Weighted mode | 233 | 0.44 | 0.9 (0.7 to 1.17) | 225 | 0.41 | 0.90 (0.71 to 1.15) |
| KC UKB | SHBG | MR Egger | 262 | 0.77 | 0.98 (0.89 to 1.09) | 254 | 0.74 | 0.98 (0.88 to 1.09) |
| KC UKB | SHBG | Weighted median | 262 | 0.54 | 1.03 (0.94 to 1.13) | 254 | 0.55 | 1.03 (0.93 to 1.14) |
| KC UKB | SHBG | IVW (fixed effect) | 262 | 0.22 | 1.05 (0.97 to 1.12) | 254 | 0.22 | 1.05 (0.97 to 1.12) |
| KC UKB | SHBG | Simple mode | 262 | 0.31 | 1.11 (0.91 to 1.37) | 254 | 0.29 | 1.12 (0.91 to 1.39) |
| KC UKB | SHBG | Weighted mode | 262 | 0.47 | 1.03 (0.95 to 1.11) | 254 | 0.63 | 1.02 (0.94 to 1.10) |
| melanoma | SHBG | MR Egger | 235 | 0.33 | 0.94 (0.84 to 1.06) | 227 | 0.26 | 0.93 (0.83 to 1.05) |

| | | | | | | | | |
|---|---|---|---|---|---|---|---|---|
| melanoma | SHBG | Weighted median | 235 | 0.68 | 0.97 (0.86 to 1.11) | 227 | 0.67 | 0.97 (0.86 to 1.10) |
| melanoma | SHBG | IVW (fixed effect) | 23 | 0.22 | 0.95 (0.88 to 1.03) | 227 | 0.25 | 0.95 (0.88 to 1.03) |
| melanoma | SHBG | Simple mode | 235 | 0.72 | 0.95 (0.72 to 1.25) | 227 | 0.75 | 0.96 (0.72 to 1.26) |
| melanoma | SHBG | Weighted mode | 235 | 0.61 | 0.97 (0.88 to 1.08) | 227 | 0.42 | 0.96 (0.86 to 1.07) |
| SCC QSKIN | SHBG | MR Egger | 234 | 0.69 | 1.1 (0.7 to 1.72) | 226 | 0.73 | 1.08 (0.69 to 1.70) |
| SCC QSKIN | SHBG | Weighted median | 234 | 0.24 | 0.75 (0.46 to 1.21) | 226 | 0.3 | 0.77 (0.47 to 1.26) |
| SCC QSKIN | SHBG | IVW (fixed effect) | 234 | 1 | 1 (0.74 to 1.36) | 226 | 0.96 | 1.01 (0.74 to 1.37) |
| SCC QSKIN | SHBG | Simple mode | 234 | 0.46 | 1.53 (0.49 to 4.72) | 226 | 0.36 | 1.72 (0.54 to 5.42) |
| SCC QSKIN | SHBG | Weighted mode | 234 | 0.7 | 0.92 (0.6 to 1.41) | 226 | 0.68 | 0.91 (0.60 to 1.39) |
| SCC UKB | SHBG | MR Egger | 262 | 0.63 | 0.96 (0.83 to 1.12) | 254 | 0.7 | 0.97 (0.83 to 1.13) |
| SCC UKB | SHBG | Weighted median | 262 | 0.73 | 0.97 (0.82 to 1.15) | 254 | 0.72 | 0.97 (0.82 to 1.14) |
| SCC UKB | SHBG | IVW (fixed effect) | 262 | 0.92 | 0.99 (0.9 to 1.1) | 254 | 0.86 | 0.99 (0.90 to 1.10) |
| SCC UKB | SHBG | Simple mode | 262 | 0.85 | 1.03 (0.73 to 1.47) | 254 | 0.82 | 1.05 (0.72 to 1.51) |
| SCC UKB | SHBG | Weighted mode | 262 | 0.56 | 0.96 (0.83 to 1.11) | 254 | 0.4 | 0.94 (0.82 to 1.08) |

## Supplementary Table 10. Estimated Multivariable MR derived marginal OR on skin cancer risk from MVMR model including totalT, freeT and MPB

| Outcome | Exposure | UKB | | QSKIN | | Meta-analysis combining UKB and QSKIN | | |
|---|---|---|---|---|---|---|---|---|
| | | Pval | Marginal OR (95% CI) | Pval | Marginal OR (95% CI) | Pval | Marginal OR (95% CI) | P-het |
| KC | freeT | 0.71 | 0.96 (0.79 to 1.17) | 0.90 | 1.03 (0.65 to 1.63) | 0.77 | 0.97 (0.81 to 1.17) | 0.79 |
| KC | MPB | 3.85E-03 | 1.16 (1.05 to 1.29) | 0.20 | 1.16 (0.92 to 1.47) | 1.55E-03 | 1.16 (1.06 to 1.28) | 0.99 |
| KC | totalT | 0.65 | 1.03 (0.90 to 1.19) | 0.44 | 1.13 (0.82 to 1.56) | 0.47 | 1.05 (0.92 to 1.19) | 0.60 |
| SCC | freeT | 0.93 | 1.01 (0.77 to 1.33) | 0.70 | 1.18 (0.51 to 2.72) | 0.84 | 1.03 (0.79 to 1.33) | 0.73 |
| SCC | MPB | 3.49E-04 | 1.29 (1.12 to 1.49) | 0.19 | 1.32 (0.87 to 2.00) | 1.39E-04 | 1.30 (1.13 to 1.48) | 0.92 |
| SCC | totalT | 0.63 | 0.95 (0.79 to 1.16) | 0.57 | 1.18 (0.66 to 2.11) | 0.78 | 0.97 (0.81 to 1.17) | 0.49 |
| BCC | freeT | 0.75 | 0.97 (0.78 to 1.19) | 0.82 | 0.93 (0.50 to 1.73) | 0.71 | 0.96 (0.79 to 1.18) | 0.91 |
| BCC | MPB | 0.02 | 1.15 (1.03 to 1.28) | 0.13 | 1.27 (0.93 to 1.73) | 5.22E-03 | 1.16 (1.04 to 1.29) | 0.54 |
| BCC | totalT | 0.46 | 1.06 (0.91 to 1.23) | 0.27 | 1.27 (0.83 to 1.96) | 0.29 | 1.08 (0.94 to 1.24) | 0.42 |
| Melanoma | freeT | - | - | - | - | 0.91 | 1.01 (0.83 to 1.23) | - |
| Melanoma | MPB | - | - | - | - | 0.27 | 0.95 (0.86 to 1.04) | - |
| Melanoma | totalT | - | - | - | - | 0.33 | 0.94 (0.82 to 1.07) | - |

P-het=Pvalue of the heterogeneity test.

## Supplementary Table 11. PheWAS summary on detected MR-PRESSO SNP outliers on a range of skin-related risk factors and disease traits

| SNP | CHR | ID (OpenGWAS) | Trait description | IA | P | SE | N | Beta | EAF |
|---|---|---|---|---|---|---|---|---|---|
| rs3847069 (EA:T; NEA:C) | 7 | ukb-d-C_OTHER_SKIN | **Other malignant neoplasms of skin** | | 9.64E-15 | 0.0005 | 361194 | -0.004 | 0.25 |
| | 7 | ukb-d-C3_SKIN | **Malignant neoplasm of skin** | | 8.54E-13 | 0.0006 | 361194 | -0.004 | 0.25 |
| | 7 | ukb-d-C_SKIN | **Cancer of skin** | | 8.54E-13 | 0.0006 | 361194 | -0.004 | 0.25 |
| | 7 | ukb-a-518 | Diagnoses - main ICD10: C44 Other malignant neoplasms of skin | | 2.93E-10 | 0.0003 | 337199 | -0.002 | 0.25 |
| | 7 | ukb-b-10248 | Type of cancer: ICD10: C44.3 Skin of other and unspecified parts of face | | 6.50E-10 | 0.0003 | 463010 | -0.002 | 0.25 |
| | 7 | ukb-a-303 | **Hair/balding pattern: Pattern 4** | YES | 1.16E-08 | 0.0016 | 154988 | 0.009 | 0.25 |
| | 7 | ukb-d-2395_1 | Hair/balding pattern: Pattern 1 | | 1.10E-06 | 0.0019 | 165649 | -0.009 | 0.25 |
| rs2669871 (EA:T; NEA:C) | 12 | ukb-d-C_OTHER_SKIN | **Other malignant neoplasms of skin** | | 2.37E-11 | 0.0005 | 361194 | -0.003 | 0.49 |
| | 12 | ukb-d-C_SKIN | **Cancer of skin** | | 8.35E-10 | 0.0005 | 361194 | -0.003 | 0.49 |
| | 12 | ukb-d-C3_SKIN | **Malignant neoplasm of skin** | | 8.35E-10 | 0.0005 | 361194 | -0.003 | 0.49 |
| | 12 | ukb-a-518 | Diagnoses - main ICD10: C44 Other malignant neoplasms of skin | | 3.60E-07 | 0.0003 | 337199 | -0.001 | 0.49 |
| rs1805007 (EA:T; NEA:C) | 16 | ukb-b-19560 | **Skin colour** | YES | 0 | 0.0019 | 456692 | -0.225 | 0.1 |
| | 16 | ukb-b-13246 | **Childhood sunburn occasions** | | 0 | 0.0035 | 346955 | 0.196 | 0.1 |
| | 16 | ukb-b-533 | **Ease of skin tanning** | YES | 0 | 0.0032 | 453065 | 0.428 | 0.1 |
| | 16 | ukb-d-1747_2 | **Hair colour (natural, before greying): Red** | YES | 0 | 0.0008 | 360270 | 0.162 | 0.1 |
| | 16 | ukb-d-1747_4 | **Hair colour (natural, before greying): Dark brown** | YES | 0 | 0.0018 | 360270 | -0.143 | 0.1 |
| | 16 | ukb-b-7422 | **Use of sun/uv protection** | | 1.50E-272 | 0.003 | 459416 | 0.104 | 0.1 |
| | 16 | ukb-d-1747_5 | **Hair colour (natural, before greying): Black** | YES | 8.18E-258 | 0.0008 | 360270 | -0.027 | 0.1 |
| | 16 | ukb-d-1747_1 | **Hair colour (natural, before greying): Blonde** | YES | 7.03E-130 | 0.0012 | 360270 | 0.03 | 0.1 |
| | 16 | ukb-d-C3_SKIN | **Malignant neoplasm of skin** | | 2.85E-80 | 0.0008 | 361194 | 0.015 | 0.1 |
| | 16 | ukb-d-C_SKIN | **Cancer of skin** | | 2.85E-80 | 0.0008 | 361194 | 0.015 | 0.1 |
| | 16 | ukb-d-C_OTHER_SKIN | Other malignant neoplasms of skin | | 3.88E-59 | 0.0007 | 361194 | 0.012 | 0.1 |
| | 16 | ukb-d-1747_6 | **Hair colour (natural, before greying): Other** | YES | 9.98E-56 | 0.0004 | 360270 | 0.007 | 0.1 |
| | 16 | ukb-d-1747_3 | **Hair colour (natural, before greying): Light brown** | YES | 5.40E-50 | 0.0019 | 360270 | -0.028 | 0.1 |
| | 16 | ukb-b-12915 | Cancer code, self-reported: malignant melanoma | | 4.40E-38 | 0.0003 | 462933 | 0.004 | 0.1 |
| | 16 | ukb-d-C3_MELANOMA_SKIN | **Malignant melanoma of skin** | | 5.24E-36 | 0.0003 | 361194 | 0.004 | 0.1 |
| | 16 | ukb-a-58 | Cancer code self-reported: malignant melanoma | | 1.68E-30 | 0.0004 | 337159 | 0.004 | 0.1 |
| | 16 | ukb-b-10787 | Standing height | | 3.30E-28 | 0.0022 | 461950 | -0.024 | 0.1 |

| | | | | | | | | | | |
|---|---|---|---|---|---|---|---|---|---|---|
| | 16 | ukb-d-C43 | Diagnoses - main ICD10: C43 Malignant melanoma of skin | | 3.35E-25 | 0.0003 | 361194 | 0.003 | 0.1 |
| | 16 | ukb-a-518 | Diagnoses - main ICD10: C44 Other malignant neoplasms of skin | | 1.30E-23 | 0.0005 | 337199 | 0.005 | 0.1 |
| | 16 | ukb-b-8837 | Cancer code, self-reported: basal cell carcinoma | | 3.50E-22 | 0.0003 | 462933 | 0.003 | 0.1 |
| | 16 | ukb-b-5945 | Relative age of first facial hair | | 1.90E-20 | 0.0022 | 204568 | 0.021 | 0.1 |
| | 16 | ukb-a-389 | Standing height | | 3.92E-19 | 0.0028 | 336474 | -0.025 | 0.1 |
| | 16 | ukb-b-2148 | **Facial ageing** | | 8.10E-19 | 0.0017 | 423999 | 0.015 | 0.1 |
| | 16 | ukb-b-660 | Number of self-reported cancers | | 7.70E-18 | 0.0011 | 462925 | 0.009 | 0.1 |
| | 16 | ukb-b-6772 | Operation code: removal of mole/skin lesion | | 1.60E-17 | 0.0004 | 462933 | 0.003 | 0.1 |
| | 16 | ukb-a-59 | Cancer code self-reported: basal cell carcinoma | | 6.67E-17 | 0.0004 | 337159 | 0.003 | 0.1 |
| | 16 | ukb-a-21 | Number of self-reported cancers | | 4.19E-15 | 0.0012 | 337154 | 0.01 | 0.1 |
| | 16 | ukb-a-299 | Relative age of first facial hair | | 2.04E-14 | 0.0026 | 151113 | 0.02 | 0.1 |
| | 16 | ukb-b-15918 | Treatment speciality of consultant (recoded): Dermatology | | 2.70E-14 | 0.0005 | 444587 | 0.004 | 0.1 |
| | 16 | ukb-b-4141 | Diagnoses - main ICD10: C44.3 Skin of other and unspecified parts of face | | 6.70E-14 | 0.0003 | 463010 | 0.002 | 0.1 |
| | 16 | ukb-b-10248 | Type of cancer: ICD10: C44.3 Skin of other and unspecified parts of face | | 2.10E-13 | 0.0004 | 463010 | 0.003 | 0.1 |
| | 16 | ukb-b-9537 | Operative procedures - main OPCS: S06.9 Unspecified other excision of lesion of skin | | 3.30E-13 | 0.0006 | 463010 | 0.004 | 0.1 |
| | 16 | ukb-b-1852 | Main speciality of consultant (recoded): Dermatology | | 9.90E-13 | 0.0005 | 461170 | 0.003 | 0.1 |
| | 16 | finn-a-L12_ACTINKERA | Actinic keratosis | | 1.52E-12 | 0.0789 | | 0.558 | 0.07 |
| | 16 | ukb-b-16855 | Cancer diagnosed by doctor | | 4.60E-12 | 0.0009 | 461311 | 0.006 | 0.1 |
| | 16 | finn-a-L12_NONIONRADISKIN | Skin changes due to chronic exposure to nonionizing radiation | | 8.02E-12 | 0.0762 | | 0.521 | 0.07 |
| | 16 | finn-a-L12_RADIATIONRELATEDSKIN | Radiation-related disorders of the skin and subcutaneous tissue | | 3.33E-11 | 0.0712 | | 0.472 | 0.07 |
| | 16 | ukb-b-2951 | Operative procedures - secondary OPCS: Z50.1 Skin of arm | | 3.40E-11 | 0.0003 | 463010 | 0.002 | 0.1 |
| | 16 | ukb-d-L12_NONIONRADISKIN | Skin changes due to chronic exposure to nonionizing radiation | | 4.27E-11 | 0.0002 | 361194 | 0.002 | 0.1 |
| | 16 | ukb-a-307 | Cancer diagnosed by doctor | | 6.44E-11 | 0.0011 | 336272 | 0.007 | 0.1 |
| | 16 | prot-a-2310 | Melanocyte protein PMEL | | 7.59E-11 | 0.0415 | 3301 | -0.27 | 0.1 |
| | 16 | ukb-d-L12_ACTINKERA | Actinic keratosis | | 1.07E-10 | 0.0002 | 361194 | 0.002 | 0.1 |
| | 16 | ukb-b-6591 | Age first had sexual intercourse | | 1.50E-10 | 0.0035 | 406457 | 0.023 | 0.1 |
| | 16 | ukb-b-5394 | Reported occurrences of cancer | | 2.10E-10 | 0.0054 | 73231 | 0.034 | 0.11 |
| | 16 | ukb-d-30260_irnt | Mean reticulocyte volume | | 3.30E-10 | 0.0039 | 344728 | -0.024 | 0.1 |
| | 16 | ukb-d-2395_1 | Hair/balding pattern: Pattern 1 | | 3.97E-10 | 0.0026 | 165649 | -0.016 | 0.1 |

| | | | | | | | | | |
|---|---|---|---|---|---|---|---|---|---|
| | 16 | ukb-b-1431 | Place of birth in UK - north co-ordinate | | 1.10E-09 | 0.0027 | 434576 | 0.016 | 0.1 |
| | 16 | ukb-b-8938 | Main speciality of consultant (recoded): Plastic surgery | | 1.10E-09 | 0.0007 | 461218 | 0.004 | 0.1 |
| | 16 | ukb-b-13285 | Treatment speciality of consultant (recoded): Plastic surgery | | 4.40E-09 | 0.0007 | 445128 | 0.004 | 0.1 |
| | 16 | ukb-b-13653 | Worry too long after embarrassment | | 6.90E-09 | 0.0017 | 443918 | 0.01 | 0.1 |
| | 16 | finn-a-C3_OTHER_SKIN | **Other malignant neoplasms of skin** | | 8.97E-09 | 0.1004 | | 0.577 | 0.07 |
| | 16 | finn-a-C3_SKIN | **Malignant neoplasm of skin** | | 8.97E-09 | 0.1004 | | 0.577 | 0.07 |
| | 16 | finn-a-C3_SKIN_EXALLC | **Malignant neoplasm of skin (ICD C excluded)** | | 1.44E-08 | 0.0999 | | 0.567 | 0.07 |
| | 16 | finn-a-C3_OTHER_SKIN_EXALLC | **Other malignant neoplasms of skin (ICD C excluded)** | | 1.44E-08 | 0.0999 | | 0.567 | 0.07 |
| rs12203592 (EA:T; NEA:C) | 6 | ukb-b-533 | **Ease of skin tanning** | YES | 0 | 0.0024 | 453065 | 0.261 | 0.22 |
| | 6 | ukb-b-13246 | **Childhood sunburn occasions** | | 0 | 0.0025 | 346955 | 0.153 | 0.22 |
| | 6 | ukb-b-19560 | **Skin colour** | YES | 0 | 0.0014 | 456692 | -0.088 | 0.22 |
| | 6 | ukb-b-2148 | **Facial ageing** | YES | 0.00E+00 | 0.0013 | 423999 | 0.049 | 0.22 |
| | 6 | ukb-b-7422 | **Use of sun/uv protection** | | 1.40E-258 | 0.0021 | 459416 | 0.073 | 0.22 |
| | 6 | ukb-b-10248 | Type of cancer: ICD10: C44.3 Skin of other and unspecified parts of face | | 1.10E-61 | 0.0003 | 463010 | 0.005 | 0.22 |
| | 6 | ukb-b-5945 | **Relative age of first facial hair** | YES | 2.00E-37 | 0.0016 | 204568 | -0.021 | 0.22 |
| | 6 | ukb-b-4141 | Diagnoses - main ICD10: C44.3 Skin of other and unspecified parts of face | | 2.20E-34 | 0.0002 | 463010 | 0.003 | 0.22 |
| | 6 | ieu-b-32 | lymphocyte cell count | | 1.37E-30 | 0.0024 | 520692 | 0.027 | 0.21 |
| | 6 | ukb-b-8837 | Cancer code, self-reported: basal cell carcinoma | | 2.70E-26 | 0.0002 | 462933 | 0.003 | 0.22 |
| | 6 | ukb-b-15685 | Type of cancer: ICD10: C44.5 Skin of trunk | | 4.70E-24 | 0.0002 | 463010 | 0.002 | 0.22 |
| | 6 | ieu-b-33 | eosinophil cell count | | 1.35E-23 | 0.0025 | 470024 | 0.025 | 0.22 |
| | 6 | ieu-b-30 | white blood cell count | | 1.61E-21 | 0.0023 | 544160 | 0.022 | 0.21 |
| | 6 | ukb-b-10817 | Particulate matter air pollution (pm2.5); 2010 | | 6.20E-17 | 0.0026 | 423796 | 0.022 | 0.21 |
| | 6 | ukb-b-9942 | Nitrogen dioxide air pollution; 2010 | | 7.00E-16 | 0.0024 | 456380 | 0.02 | 0.22 |
| | 6 | ukb-b-3169 | Number of vehicles in household | | 1.90E-15 | 0.0021 | 459696 | -0.017 | 0.22 |
| | 6 | ukb-b-12417 | Nitrogen oxides air pollution; 2010 | | 3.20E-15 | 0.0025 | 456380 | 0.019 | 0.22 |
| | 6 | ukb-b-8416 | Type of cancer: ICD10: C44.9 Malignant neoplasm of skin, unspecified | | 6.60E-15 | 0.0002 | 463010 | 0.002 | 0.22 |
| | 6 | ukb-b-5620 | Nitrogen dioxide air pollution; 2006 | | 9.90E-15 | 0.0024 | 456380 | 0.019 | 0.22 |
| | 6 | ukb-b-660 | Number of self-reported cancers | | 1.80E-13 | 0.0008 | 462925 | 0.006 | 0.22 |
| | 6 | ukb-b-13184 | Types of physical activity in last 4 weeks: Heavy DIY (eg: weeding, lawn mowing, carpentry, digging) | | 6.50E-13 | 0.0012 | 460376 | -0.009 | 0.22 |
| | 6 | ukb-b-10011 | Townsend deprivation index at recruitment | | 1.70E-12 | 0.0024 | 462464 | 0.017 | 0.22 |

| | | | | | | | | | |
|---|---|---|---|---|---|---|---|---|---|
| | 6 | ebi-a-GCST004606 | Eosinophil counts | | 2.16E-12 | 0.0043 | 172275 | 0.03 | 0.22 |
| | 6 | ukb-b-1260 | Spells in hospital | | 3.50E-12 | 0.0024 | 303963 | 0.017 | 0.22 |
| | 6 | ebi-a-GCST004624 | Sum eosinophil basophil counts | | 3.76E-12 | 0.0043 | 171771 | 0.03 | 0.22 |
| | 6 | ukb-b-11231 | Diagnoses - secondary ICD10: Z85.8 Personal history of malignant neoplasms of other organs and systems | | 1.20E-11 | 0.0002 | 463010 | 0.001 | 0.22 |
| | 6 | ukb-b-2618 | Nitrogen dioxide air pollution; 2005 | | 3.60E-11 | 0.0024 | 456380 | 0.016 | 0.22 |
| | 6 | ukb-b-11312 | Particulate matter air pollution (pm2.5) absorbance; 2010 | | 1.20E-10 | 0.0026 | 423796 | 0.017 | 0.21 |
| | 6 | ukb-b-16878 | Alcohol usually taken with meals | | 1.30E-10 | 0.0016 | 235645 | -0.01 | 0.22 |
| | 6 | ukb-b-15918 | Treatment speciality of consultant (recoded): Dermatology | | 1.40E-10 | 0.0004 | 444587 | 0.002 | 0.22 |
| | 6 | prot-a-1255 | Transmembrane glycoprotein NMB | | 1.45E-10 | 0.0317 | 3301 | -0.203 | 0.2 |
| | 6 | ukb-b-20544 | Nervous feelings | | 1.50E-10 | 0.0011 | 450700 | 0.007 | 0.22 |
| | 6 | ebi-a-GCST004627 | Lymphocyte counts | | 4.56E-10 | 0.0044 | 171643 | 0.027 | 0.22 |
| | 6 | ukb-b-969 | Time spend outdoors in summer | | 4.80E-10 | 0.0023 | 419314 | -0.014 | 0.22 |
| | 6 | ukb-b-1996 | Salad / raw vegetable intake | | 1.30E-09 | 0.0017 | 435435 | -0.01 | 0.22 |
| | 6 | prot-a-1256 | Transmembrane glycoprotein NMB | | 1.78E-09 | 0.0317 | 3301 | -0.191 | 0.2 |
| | 6 | ukb-b-4630 | Neuroticism score | | 1.90E-09 | 0.0088 | 374323 | 0.053 | 0.22 |
| | 6 | ukb-b-1285 | Nitrogen dioxide air pollution; 2007 | | 1.90E-09 | 0.0024 | 456380 | 0.014 | 0.22 |
| | 6 | finn-a-L12_ACTINKERA | Actinic keratosis | | 3.74E-09 | 0.1204 | | 0.71 | 0.03 |
| | 6 | ieu-b-69 | sepsis | | 3.90E-09 | 0.0169 | 462918 | 0.047 | 0.22 |
| | 6 | finn-a-L12_NONIONRADISKIN | Skin changes due to chronic exposure to nonionizing radiation | | 4.30E-09 | 0.1165 | | 0.684 | 0.03 |
| | 6 | ukb-b-698 | How are people in household related to participant: Husband, wife or partner | | 5.90E-09 | 0.0011 | 459600 | -0.006 | 0.22 |
| | 6 | ukb-b-4263 | Number of full brothers | | 1.60E-08 | 0.0019 | 455347 | 0.011 | 0.22 |
| | 6 | ieu-b-34 | neutrophil cell count | | 1.66E-08 | 0.0024 | 515057 | 0.014 | 0.21 |
| | 6 | ukb-b-6306 | Overall health rating | | 1.80E-08 | 0.0018 | 460844 | 0.01 | 0.22 |
| | 6 | finn-a-L12_RADIATIONRELATEDSKIN | Radiation-related disorders of the skin and subcutaneous tissue | | 2.08E-08 | 0.1091 | | 0.612 | 0.03 |
| | 6 | ebi-a-GCST006948 | Feeling nervous | | 2.46E-08 | 0.0028 | 373121 | 0.016 | 0.21 |
| | 6 | ukb-b-11582 | Diagnoses - main ICD10: K62.5 Haemorrhage of anus and rectum | | 2.90E-08 | 0.0004 | 463010 | 0.002 | 0.22 |
| | 6 | ukb-b-16751 | Diagnoses - secondary ICD10: J44.9 Chronic obstructive pulmonary disease, unspecified | | 3.10E-08 | 0.0002 | 463010 | 0.001 | 0.22 |
| | 6 | ukb-b-3460 | Alcohol intake versus 10 years previously | | 3.30E-08 | 0.0019 | 428117 | 0.01 | 0.22 |

IA=SNP Instruments available (obtainable from GWAS data on exposure risk factors).

## Supplementary Table 12. Univariate MR association between skin/hair colour and MPB score (incl alt models)

| Outcome | Exposure | MR method | *Original estimates* | | | | *Removing the IRF4 variant* | | |
|---|---|---|---|---|---|---|---|---|---|
| | | | **SNPs** | **beta** | **se** | **Pvalue** | **beta** | **se** | **Pvalue** |
| MPB | Hair colour | MR Egger | 139 | 0.177 | 0.028 | 3.35E-09 | 0.025 | 0.041 | 0.55 |
| | | Weighted median | 139 | 0.252 | 0.016 | 1.97E-57 | 0.011 | 0.022 | 0.62 |
| | | Inverse variance weighted | 139 | 0.128 | 0.025 | 1.90E-07 | -0.001 | 0.031 | 0.98 |
| | | Simple mode | 139 | 0.007 | 0.059 | 0.90 | 0.007 | 0.059 | 0.91 |
| | | Weighted mode | 139 | 0.250 | 0.021 | 1.49E-22 | 0.007 | 0.018 | 0.71 |
| MPB | Skin colour | MR Egger | 39 | -0.501 | 0.091 | 2.80E-06 | -0.025 | 0.088 | 0.78 |
| | | Weighted median | 39 | -0.143 | 0.064 | 0.03 | 0.006 | 0.049 | 0.90 |
| | | Inverse variance weighted | 39 | -0.284 | 0.072 | 8.31E-05 | 0.033 | 0.055 | 0.54 |
| | | Simple mode | 39 | -0.014 | 0.079 | 0.86 | -0.012 | 0.076 | 0.87 |
| | | Weighted mode | 39 | -0.708 | 0.044 | 1.05E-18 | -0.019 | 0.048 | 0.69 |

Instruments defined at SNP association p-value < 1e-10 with exposure.
MR beta reflect the change in MPB SD units (positive indicate increased rates of balding) per 1 SD unit change in the underlying pigmentation variable (skin:increased degrees of tanning; hair: increased brightness (black - red) of hair).

## Supplementary Table 13. Estimated Multivariable MR derived marginal OR on skin cancer risk from MVMR model including totalT, freeT, hair colour and MPB

| Outcome | Exposure | UKB | | QSKIN | | Meta-analysis combining UKB and QSKIN | | |
|---|---|---|---|---|---|---|---|---|
| | | **Pval** | **Marginal OR (95% CI)** | **Pval** | **Marginal OR (95% CI)** | **Pval** | **Marginal OR (95% CI)** | **P-het** |
| KC | freeT | 0.53 | 0.95 (0.8 to 1.13) | 0.59 | 0.89 (0.58 to 1.37) | 0.43 | 0.94 (0.80 to 1.10) | 0.79 |
| KC | hair colour | 7.49E-04 | 1.18 (1.07 to 1.3) | 0.01 | 1.34 (1.07 to 1.7) | 4.79E-05 | 1.20 (1.10 to 1.31) | 0.31 |
| KC | MPB | 8.06E-04 | 1.18 (1.07 to 1.3) | 0.11 | 1.21 (0.96 to 1.52) | 2.11E-04 | 1.18 (1.08 to 1.29) | 0.85 |
| KC | totalT | 0.53 | 1.04 (0.92 to 1.17) | 0.47 | 1.12 (0.83 to 1.5) | 0.39 | 1.05 (0.94 to 1.17) | 0.67 |
| SCC | freeT | 0.91 | 1.01 (0.8 to 1.29) | 0.83 | 0.92 (0.43 to 1.98) | 0.97 | 1.00 (0.80 to 1.26) | 0.82 |
| SCC | hair colour | 6.72E-04 | 1.26 (1.1 to 1.44) | 0.01 | 1.71 (1.12 to 2.61) | 6.67E-05 | 1.30 (1.14 to 1.47) | 0.18 |
| SCC | MPB | 2.21E-04 | 1.29 (1.13 to 1.47) | 0.06 | 1.49 (0.98 to 2.25) | 4.23E-05 | 1.30 (1.15 to 1.48) | 0.51 |
| SCC | totalT | 0.59 | 0.96 (0.81 to 1.13) | 0.63 | 1.14 (0.67 to 1.92) | 0.72 | 0.97 (0.83 to 1.14) | 0.54 |
| BCC | freeT | 0.52 | 0.94 (0.78 to 1.13) | 0.23 | 0.71 (0.4 to 1.25) | 0.32 | 0.91 (0.76 to 1.09) | 0.35 |
| BCC | hair colour | 2.91E-03 | 1.17 (1.06 to 1.3) | 0.02 | 1.46 (1.07 to 1.99) | 3.26E-04 | 1.20 (1.09 to 1.32) | 0.18 |
| BCC | MPB | 3.83E-03 | 1.17 (1.05 to 1.3) | 0.03 | 1.39 (1.03 to 1.89) | 6.02E-04 | 1.19 (1.08 to 1.31) | 0.28 |
| BCC | totalT | 0.30 | 1.07 (0.94 to 1.22) | 0.36 | 1.2 (0.81 to 1.76) | 0.21 | 1.08 (0.96 to 1.23) | 0.60 |
| | | | | | | **GenoMEL Phase 2 meta-analysis** | | |
| Melanoma | freeT | - | - | - | - | 0.73 | 0.97 (0.79 to 1.18) | - |
| Melanoma | hair colour | - | - | - | - | 0.98 | 1 (0.96 to 1.04) | - |
| Melanoma | MPB | - | - | - | - | 0.34 | 0.95 (0.86 to 1.05) | - |
| Melanoma | totalT | - | - | - | - | 0.99 | 1 (0.88 to 1.14) | - |

P-het=Pvalue of the Heterogeneity test.

## Supplementary Table 14. Estimated Multivariable MR derived marginal OR on skin cancer risk from MVMR model including totalT, freeT, hair colour, skin colour and MPB

| Outcome | Exposure | UKB | | QSKIN | | Meta-analysis combining UKB and QSKIN | | |
|---|---|---|---|---|---|---|---|---|
| | | **Pval** | **Marginal OR (95% CI)** | **Pval** | **Marginal OR (95% CI)** | **Pval** | **Marginal OR (95% CI)** | **P-het** |
| KC | freeT | 0.53 | 0.96 (0.85 to 1.09) | 0.67 | 0.92 (0.65 to 1.32) | 0.46 | 0.96 (0.85 to 1.08) | 0.85 |
| KC | hair colour | 3.72E-33 | 1.59 (1.47 to 1.72) | 1.31E-16 | 2.4 (1.95 to 2.95) | <1E-100 | 1.67 (1.56 to 1.79) | 2.70E-04 |
| *KC* | *MPB* | *0.10* | *1.06 (0.99 to 1.14)* | *0.85* | *0.98 (0.81 to 1.19)* | *0.14* | *1.05 (0.98 to 1.13)* | *0.45* |
| KC | skin colour | 5.40E-106 | 0.24 (0.21 to 0.27) | 1.11E-55 | 0.06 (0.04 to 0.09) | <1E-100 | 0.20 (0.18 to 0.23) | 2.90E-13 |
| KC | totalT | 0.50 | 1.03 (0.94 to 1.13) | 0.52 | 1.08 (0.85 to 1.39) | 0.40 | 1.04 (0.95 to 1.13) | 0.71 |
| SCC | freeT | 0.90 | 1.01 (0.82 to 1.25) | 0.99 | 1.01 (0.52 to 1.96) | 0.90 | 1.01 (0.83 to 1.24) | 0.98 |
| SCC | hair colour | 4.57E-17 | 1.71 (1.51 to 1.94) | 2.92E-14 | 4.78 (3.19 to 7.15) | <1E-100 | 1.87 (1.66 to 2.11) | 1.86E-06 |
| *SCC* | *MPB* | *0.01* | *1.17 (1.04 to 1.31)* | *0.61* | *1.1 (0.76 to 1.58)* | *0.01* | *1.16 (1.04 to 1.30)* | *0.77* |
| SCC | skin colour | 3.53E-39 | 0.24 (0.2 to 0.3) | 5.07E-40 | 0.01 (0 to 0.02) | <1E-100 | 0.18 (0.15 to 0.23) | 0.00E+00 |
| SCC | totalT | 0.50 | 0.95 (0.82 to 1.1) | 0.72 | 1.09 (0.69 to 1.72) | 0.59 | 0.96 (0.84 to 1.11) | 0.59 |
| BCC | freeT | 0.57 | 0.96 (0.83 to 1.1) | 0.24 | 0.75 (0.47 to 1.21) | 0.38 | 0.94 (0.82 to 1.08) | 0.33 |
| BCC | hair colour | 7.20E-30 | 1.63 (1.49 to 1.77) | 5.66E-16 | 3.18 (2.4 to 4.2) | <1E-50 | 1.72 (1.58 to 1.86) | 6.85E-06 |
| *BCC* | *MPB* | *0.29* | *1.04 (0.96 to 1.13)* | *0.55* | *1.08 (0.84 to 1.4)* | *0.24* | *1.05 (0.97 to 1.13)* | *0.80* |
| BCC | skin colour | 4.56E-102 | 0.21 (0.19 to 0.25) | 9.41E-51 | 0.03 (0.02 to 0.04) | <1E-100 | 0.18 (0.16 to 0.21) | 2.22E-16 |
| BCC | totalT | 0.24 | 1.06 (0.96 to 1.17) | 0.39 | 1.15 (0.83 to 1.6) | 0.17 | 1.07 (0.97 to 1.17) | 0.63 |
| Melanoma | freeT | - | - | - | - | 0.98 | 1 (0.82 to 1.21) | - |
| Melanoma | hair colour | - | - | - | - | 1.29E-06 | 1.19 (1.11 to 1.28) | - |
| *Melanoma* | *MPB* | - | - | - | - | *0.08* | *0.91 (0.82 to 1.01)* | - |
| Melanoma | skin colour | - | - | - | - | 5.82E-09 | 0.5 (0.4 to 0.64) | - |
| Melanoma | totalT | - | - | - | - | 0.69 | 1.03 (0.9 to 1.16) | - |

Estimates for MPB are shown in italic font. KC = any BCC or SCC.

## Supplementary Table 15. Sensitivity analyses assessing the influence of the 4 detected MR-PRESSO SNP outliers on MVMR marginal OR estimates

| Outcome | Trait | Before removing SNP-outlier | | After removing SNP-outlier | |
|---|---|---|---|---|---|
| | | **Pvalue** | **marginal OR (95% CI)** | **Pvalue** | **marginal OR (95% CI)** |
| KC | hair colour | 3.35E-45 | 1.67 (1.56 to 1.80) | 1.36E-10 | 1.56 (1.36 to 1.79) |
| *KC* | *MPB* | *0.16* | *1.05 (0.98 to 1.12)* | *0.13* | *1.05 (0.98 to 1.13)* |
| KC | skin colour | 8.25E-149 | 0.20 (0.18 to 0.23) | 1.09E-31 | 0.22 (0.17 to 0.28) |
| KC | totalT | 0.70 | 1.01 (0.94 to 1.09) | 0.66 | 1.02 (0.94 to 1.10) |
| SCC | hair colour | 8.15E-25 | 1.87 (1.66 to 2.11) | 1.47E-03 | 1.45 (1.15 to 1.81) |
| *SCC* | *MPB* | *0.01* | *1.16 (1.04 to 1.29)* | *0.02* | *1.15 (1.02 to 1.28)* |
| SCC | skin colour | 3.86E-61 | 0.18 (0.15 to 0.22) | 2.06E-08 | 0.29 (0.19 to 0.45) |
| SCC | totalT | 0.61 | 0.97 (0.85 to 1.10) | 0.61 | 0.97 (0.86 to 1.10) |
| BCC | hair colour | 1.14E-39 | 1.72 (1.59 to 1.86) | 5.59E-10 | 1.62 (1.39 to 1.88) |
| *BCC* | *MPB* | *0.27* | *1.04 (0.97 to 1.13)* | *0.21* | *1.05 (0.97 to 1.13)* |
| BCC | skin colour | 5.96E-136 | 0.18 (0.16 to 0.21) | 4.65E-31 | 0.18 (0.14 to 0.24) |
| BCC | totalT | 0.39 | 1.04 (0.95 to 1.13) | 0.35 | 1.04 (0.96 to 1.13) |
| Melanoma | hair colour | 9.41E-07 | 1.19 (1.11 to 1.28) | 1.17E-15 | 1.57 (1.41 to 1.76) |
| *Melanoma* | *MPB* | *0.08* | *0.91 (0.82 to 1.01)* | *0.30* | *0.95 (0.86 to 1.05)* |
| Melanoma | skin colour | 6.71E-09 | 0.51 (0.4 to 0.64) | 1.07E-17 | 0.2 (0.14 to 0.29) |
| Melanoma | totalT | 0.81 | 1.01 (0.91 to 1.14) | 0.34 | 0.95 (0.85 to 1.06) |

Estimates for MPB are shown in italic font. KC = any BCC or SCC.

## Supplementary Table 16. Estimated heterogeneity among SNP estimates on MPB and skin cancer

| Outcome | Exposure | Model | Original estimate | | | After removing SNP-outliers identified in MR-PRESSO | | |
|---|---|---|---|---|---|---|---|---|
| | | | **Cochran Q** | **Q_df** | **Q_pval** | **Cochran Q** | **Q_df** | **Q_pval** |
| KC UKB* | MPB | MR Egger | 1154.7 | 470 | 2.05E-59 | 561.2 | 466 | 1.58E-03 |
| | | IVW | 1156.1 | 471 | 2.09E-59 | 563.6 | 467 | 1.41E-03 |
| KC QSKIN | MPB | MR Egger | 650.6 | 468 | 4.35E-08 | 457.1 | 464 | 5.82E-01 |
| | | IVW | 658.9 | 469 | 1.52E-08 | 459.0 | 465 | 5.70E-01 |
| SCC UKB | MPB | MR Egger | 680.1 | 470 | 6.92E-10 | 476.0 | 466 | 3.65E-01 |
| | | IVW | 680.6 | 471 | 7.71E-10 | 480.8 | 467 | 3.20E-01 |
| SCC QSKIN | MPB | MR Egger | 662.0 | 469 | 9.46E-09 | 503.9 | 465 | 1.03E-01 |
| | | IVW | 668.0 | 470 | 4.62E-09 | 505.1 | 466 | 1.02E-01 |
| BCC UKB* | MPB | MR Egger | 1149.3 | 470 | 1.02E-58 | 591.9 | 466 | 6.56E-05 |
| | | IVW | 1152.8 | 471 | 5.54E-59 | 592.5 | 467 | 6.97E-05 |
| BCC QSKIN | MPB | MR Egger | 677.2 | 469 | 9.11E-10 | 504.2 | 465 | 1.02E-01 |
| | | IVW | 681.7 | 470 | 5.41E-10 | 504.7 | 466 | 1.05E-01 |
| Melanoma | MPB | MR Egger | 626.6 | 469 | 1.41E-06 | 616.5 | 466 | 3.36E-06 |
| | | IVW | 626.6 | 470 | 1.64E-06 | 616.5 | 467 | 3.89E-06 |

Cochran Q=The Cochran Q test statistics. Q_df =degree of freedom for the Cochran Q test statistics. Q_pval=Pvalue of the Cochran Q test statistics.

## Supplementary Table 17. Estimated heterogeneity among SNP estimates on MPB and skin cancer using MPB variants with p<1e-5 in the independent UKB subset.

| Outcome | Exposure | Model | Original estimate | | | After removing SNP-outliers identified in MR-PRESSO | | |
|---|---|---|---|---|---|---|---|---|
| | | | Cochran Q | Q_df | Q_pval | Cochran Q | Q_df | Q_pval |
| KC UKB | MPB (P<1e-5 only) | MR Egger | 958.8 | 285 | 8.19E-74 | 370.3 | 281 | 2.75E-04 |
| | | IVW | 965.6 | 286 | 1.39E-74 | 370.4 | 282 | 3.13E-04 |
| KC QSKIN | MPB (P<1e-5 only) | MR Egger | 468.6 | 284 | 3.12E-11 | 274.1 | 280 | 0.59 |
| | | IVW | 471.0 | 285 | 2.51E-11 | 274.1 | 281 | 0.60 |
| SCC UKB | MPB (P<1e-5 only) | MR Egger | 519.8 | 285 | 6.41E-16 | 315.9 | 281 | 0.07 |
| | | IVW | 519.8 | 286 | 8.62E-16 | 318.8 | 282 | 0.06 |
| SCC QSKIN | MPB (P<1e-5 only) | MR Egger | 469.8 | 285 | 3.22E-11 | 313.0 | 281 | 0.09 |
| | | IVW | 476.4 | 286 | 1.09E-11 | 314.5 | 282 | 0.09 |
| BCC UKB | MPB (P<1e-5 only) | MR Egger | 938.5 | 285 | 1.04E-70 | 386.4 | 281 | 2.96E-05 |
| | | IVW | 949.0 | 286 | 4.72E-72 | 386.5 | 282 | 3.46E-05 |
| BCC QSKIN | MPB (P<1e-5 only) | MR Egger | 477.0 | 285 | 7.31E-12 | 304.4 | 281 | 0.16 |
| | | IVW | 480.1 | 286 | 5.08E-12 | 304.5 | 282 | 0.17 |
| Melanoma | MPB (P<1e-5 only) | MR Egger | 391.7 | 284 | 2.29E-05 | 381.9 | 281 | 5.68E-05 |
| | | IVW | 392.0 | 285 | 2.62E-05 | 381.9 | 282 | 6.68E-05 |

Cochran Q=The Cochran Q test statistics. Q_df =degree of freedom for the Cochran Q test statistics. Q_pval=Pvalue of the Cochran Q test statistics. MPB (P<1e-5 only)=The analysis using MPB variants (SNP instruments) with association p-value<1e-5 in the MPB GWAS conducted on the independent UKB subset (see methods).

## Supplementary Table 18. Estimated MR ORs for per 1 SD change increase in genetically predicted MPB score on skin cancer risk after removal of (n=4) pleiotropic SNP outliers

| outcome | exposure | method | All variants | | | Variants with p<1e-5 in subset | | |
|---|---|---|---|---|---|---|---|---|
| | | | nsnp | pval | OR | nsnp | pval | OR |
| KC UKB | MPB (outliers removed) | MR Egger | 468 | 0.72 | 1.02 (0.92 to 1.14) | 283 | 0.49 | 1.05 (0.92 to 1.20) |
| KC UKB | MPB (outliers removed) | Weighted median | 468 | 0.29 | 1.05 (0.96 to 1.15) | 283 | 0.3 | 1.05 (0.96 to 1.15) |
| KC UKB | MPB (outliers removed) | IVW (fixed effect) | 468 | 3.43E-03 | 1.09 (1.03 to 1.15) | 283 | 0.04 | 1.07 (1.00 to 1.14) |
| KC UKB | MPB (outliers removed) | Simple mode | 468 | 0.65 | 1.05 (0.84 to 1.31) | 283 | 0.88 | 1.02 (0.80 to 1.29) |
| KC UKB | MPB (outliers removed) | Weighted mode | 468 | 0.91 | 0.99 (0.89 to 1.11) | 283 | 0.95 | 1.00 (0.87 to 1.13) |
| SCC UKB | MPB (outliers removed) | MR Egger | 468 | 0.88 | 1.01 (0.85 to 1.21) | 283 | 0.98 | 1.00 (0.81 to 1.25) |
| SCC UKB | MPB (outliers removed) | Weighted median | 468 | 0.01 | 1.24 (1.06 to 1.45) | 283 | 0.01 | 1.24 (1.05 to 1.47) |
| SCC UKB | MPB (outliers removed) | IVW (fixed effect) | 468 | 1.66E-04 | 1.20 (1.09 to 1.32) | 283 | 2.85E-03 | 1.17 (1.06 to 1.31) |
| SCC UKB | MPB (outliers removed) | Simple mode | 468 | 0.1 | 1.35 (0.94 to 1.93) | 283 | 0.1 | 1.38 (0.94 to 2.04) |
| SCC UKB | MPB (outliers removed) | Weighted mode | 468 | 0.1 | 1.20 (0.97 to 1.48) | 283 | 0.1 | 1.22 (0.96 to 1.55) |
| BCC QSKIN | MPB (outliers removed) | MR Egger | 467 | 0.85 | 1.04 (0.68 to 1.61) | 283 | 0.96 | 1.01 (0.61 to 1.68) |
| BCC QSKIN | MPB (outliers removed) | Weighted median | 467 | 0.65 | 0.92 (0.63 to 1.34) | 283 | 0.77 | 0.94 (0.64 to 1.40) |
| BCC QSKIN | MPB (outliers removed) | IVW (fixed effect) | 467 | 0.46 | 0.92 (0.73 to 1.15) | 283 | 0.7 | 0.95 (0.75 to 1.22) |
| BCC QSKIN | MPB (outliers removed) | Simple mode | 467 | 0.06 | 0.41 (0.16 to 1.05) | 283 | 0.21 | 0.56 (0.23 to 1.37) |
| BCC QSKIN | MPB (outliers removed) | Weighted mode | 467 | 0.39 | 0.81 (0.51 to 1.30) | 283 | 0.43 | 0.80 (0.46 to 1.39) |
| BCC UKB | MPB (outliers removed) | MR Egger | 468 | 0.59 | 1.03 (0.91 to 1.17) | 283 | 0.37 | 1.07 (0.92 to 1.25) |
| BCC UKB | MPB (outliers removed) | Weighted median | 468 | 0.34 | 1.05 (0.95 to 1.15) | 283 | 0.4 | 1.05 (0.94 to 1.16) |
| BCC UKB | MPB (outliers removed) | IVW (fixed effect) | 468 | 0.03 | 1.07 (1.00 to 1.14) | 283 | 0.16 | 1.05 (0.98 to 1.13) |
| BCC UKB | MPB (outliers removed) | Simple mode | 468 | 0.52 | 1.09 (0.84 to 1.40) | 283 | 0.66 | 1.06 (0.82 to 1.37) |
| BCC UKB | MPB (outliers removed) | Weighted mode | 468 | 0.84 | 0.99 (0.86 to 1.13) | 283 | 0.82 | 0.98 (0.85 to 1.14) |
| KC QSKIN | MPB (outliers removed) | MR Egger | 466 | 0.67 | 1.07 (0.79 to 1.44) | 282 | 0.73 | 0.94 (0.66 to 1.34) |
| KC QSKIN | MPB (outliers removed) | Weighted median | 466 | 0.61 | 0.93 (0.70 to 1.23) | 282 | 0.68 | 0.94 (0.71 to 1.25) |
| KC QSKIN | MPB (outliers removed) | IVW (fixed effect) | 466 | 0.14 | 0.89 (0.76 to 1.04) | 282 | 0.48 | 0.94 (0.79 to 1.11) |
| KC QSKIN | MPB (outliers removed) | Simple mode | 466 | 0.42 | 0.77 (0.41 to 1.45) | 282 | 0.79 | 0.91 (0.46 to 1.79) |
| KC QSKIN | MPB (outliers removed) | Weighted mode | 466 | 0.91 | 0.98 (0.67 to 1.42) | 282 | 0.99 | 1.00 (0.65 to 1.55) |

| | | | | | | | | |
|---|---|---|---|---|---|---|---|---|
| SCC QSKIN | MPB (outliers removed) | MR Egger | 467 | 0.67 | 1.13 (0.63 to 2.02) | 283 | 0.5 | 1.27 (0.64 to 2.53) |
| SCC QSKIN | MPB (outliers removed) | Weighted median | 467 | 0.83 | 1.06 (0.64 to 1.74) | 283 | 0.75 | 1.09 (0.65 to 1.83) |
| SCC QSKIN | MPB (outliers removed) | IVW (fixed effect) | 467 | 0.35 | 0.87 (0.64 to 1.18) | 283 | 0.51 | 0.89 (0.64 to 1.24) |
| SCC QSKIN | MPB (outliers removed) | Simple mode | 467 | 0.74 | 0.79 (0.19 to 3.20) | 283 | 0.45 | 0.60 (0.16 to 2.26) |
| SCC QSKIN | MPB (outliers removed) | Weighted mode | 467 | 0.76 | 1.13 (0.51 to 2.51) | 283 | 0.63 | 1.23 (0.54 to 2.78) |
| melanoma | MPB (outliers removed) | MR Egger | 466 | 0.74 | 0.97 (0.84 to 1.14) | 283 | 0.73 | 0.97 (0.81 to 1.16) |
| melanoma | MPB (outliers removed) | Weighted median | 466 | 0.59 | 0.97 (0.86 to 1.09) | 283 | 0.61 | 0.97 (0.85 to 1.10) |
| melanoma | MPB (outliers removed) | IVW (fixed effect) | 466 | 0.39 | 0.96 (0.88 to 1.05) | 283 | 0.39 | 0.96 (0.88 to 1.05) |
| melanoma | MPB (outliers removed) | Simple mode | 466 | 0.05 | 1.32 (1.00 to 1.76) | 283 | 0.31 | 1.16 (0.87 to 1.57) |
| melanoma | MPB (outliers removed) | Weighted mode | 466 | 0.3 | 1.09 (0.93 to 1.27) | 283 | 0.43 | 1.08 (0.89 to 1.30) |

Variants with P<1e-5 in subset=The analysis using MPB variants (SNP instruments) with association p-value<1e-5 in the MPB GWAS conducted on the independent UKB subset (see methods).

## Supplementary Table 19. Estimated (meta-analysed) MR ORs for per 1 SD change increase in genetically predicted MPB score on skin cancer risk stratified by major body site categories.

| Cancer | Category | MR Method | Original estimate | | | After removal of SNP-outliers | |
|---|---|---|---|---|---|---|---|
| | | | SNPs | Pvalue | OR | Pvalue | OR |
| BCC | Head and Neck | MR Egger | 442 | 0.06 | 1.16 (0.99 to 1.35) | 0.10 | 1.14 (0.97 to 1.33) |
| | | Weighted median | 442 | 0.26 | 1.08 (0.94 to 1.23) | 0.28 | 1.08 (0.94 to 1.23) |
| | | Inverse variance weighted | 442 | 0.05 | 1.08 (1.00 to 1.17) | 0.08 | 1.08 (0.99 to 1.17) |
| | | Simple mode | 442 | 0.76 | 1.05 (0.77 to 1.43) | 0.83 | 1.04 (0.74 to 1.44) |
| | | Weighted mode | 442 | 0.39 | 1.08 (0.91 to 1.28) | 0.40 | 1.08 (0.90 to 1.29) |
| | Upper limb | MR Egger | 441 | 0.46 | 1.14 (0.81 to 1.61) | 0.80 | 1.05 (0.74 to 1.48) |
| | | Weighted median | 441 | 0.80 | 1.04 (0.76 to 1.42) | 0.93 | 0.99 (0.73 to 1.33) |
| | | Inverse variance weighted | 441 | 0.77 | 0.97 (0.81 to 1.17) | 0.43 | 0.93 (0.77 to 1.12) |
| | | Simple mode | 441 | 0.79 | 1.11 (0.51 to 2.42) | 0.90 | 1.05 (0.50 to 2.22) |
| | | Weighted mode | 441 | 0.34 | 1.23 (0.80 to 1.89) | 0.60 | 1.13 (0.71 to 1.79) |
| | Trunk | MR Egger | 441 | 0.10 | 1.23 (0.96 to 1.58) | 0.23 | 1.17 (0.91 to 1.50) |
| | | Weighted median | 441 | 0.06 | 1.24 (0.99 to 1.56) | 0.10 | 1.22 (0.96 to 1.54) |
| | | Inverse variance weighted | 441 | 0.18 | 1.10 (0.96 to 1.25) | 0.28 | 1.08 (0.94 to 1.23) |
| | | Simple mode | 441 | 0.88 | 1.04 (0.61 to 1.79) | 0.97 | 1.01 (0.59 to 1.72) |
| | | Weighted mode | 441 | 0.21 | 1.22 (0.89 to 1.66) | 0.28 | 1.17 (0.88 to 1.56) |
| | Lower limb | MR Egger | 441 | 0.40 | 1.24 (0.75 to 2.06) | 0.77 | 1.08 (0.65 to 1.81) |
| | | Weighted median | 441 | 0.73 | 0.92 (0.59 to 1.45) | 0.60 | 0.89 (0.57 to 1.39) |
| | | Inverse variance weighted | 441 | 0.95 | 0.99 (0.76 to 1.30) | 0.69 | 0.95 (0.72 to 1.24) |
| | | Simple mode | 441 | 0.61 | 0.75 (0.24 to 2.28) | 0.60 | 0.75 (0.26 to 2.21) |
| | | Weighted mode | 441 | 0.83 | 0.94 (0.51 to 1.71) | 0.70 | 0.89 (0.49 to 1.62) |
| SCC | Head and Neck | MR Egger | 441 | 0.99 | 1.00 (0.77 to 1.29) | 0.74 | 0.96 (0.74 to 1.24) |
| | | Weighted median | 441 | 0.30 | 0.89 (0.71 to 1.11) | 0.26 | 0.87 (0.69 to 1.10) |
| | | Inverse variance weighted | 441 | 0.29 | 0.93 (0.81 to 1.06) | 0.22 | 0.92 (0.80 to 1.05) |
| | | Simple mode | 441 | 0.79 | 0.93 (0.52 to 1.65) | 0.76 | 0.92 (0.54 to 1.57) |
| | | Weighted mode | 441 | 0.69 | 0.94 (0.69 to 1.28) | 0.68 | 0.94 (0.68 to 1.29) |
| | Upper limb | MR Egger | 441 | 0.16 | 1.41 (0.87 to 2.31) | 0.39 | 1.24 (0.76 to 2.04) |
| | | Weighted median | 441 | 0.16 | 1.37 (0.88 to 2.13) | 0.23 | 1.31 (0.84 to 2.03) |
| | | Inverse variance weighted | 441 | 0.54 | 1.08 (0.84 to 1.41) | 0.88 | 1.02 (0.78 to 1.32) |
| | | Simple mode | 441 | 0.98 | 0.98 (0.34 to 2.86) | 0.90 | 0.93 (0.30 to 2.86) |
| | | Weighted mode | 441 | 0.07 | 1.82 (0.96 to 3.46) | 0.11 | 1.70 (0.89 to 3.24) |
| | Trunk | MR Egger | 441 | 0.36 | 0.77 (0.44 to 1.34) | 0.16 | 0.67 (0.38 to 1.17) |

| | | | | | | | |
|---|---|---|---|---|---|---|---|
| | | Weighted median | 441 | 0.91 | 1.03 (0.64 to 1.66) | 0.91 | 0.97 (0.59 to 1.59) |
| | | Inverse variance weighted | 441 | 0.76 | 1.05 (0.78 to 1.41) | 1.00 | 1.00 (0.74 to 1.35) |
| | | Simple mode | 441 | 0.50 | 1.53 (0.44 to 5.29) | 0.51 | 1.53 (0.43 to 5.47) |
| | | Weighted mode | 441 | 0.79 | 0.91 (0.47 to 1.79) | 0.63 | 0.84 (0.42 to 1.70) |
| | Lower limb | MR Egger | 441 | 0.20 | 1.56 (0.79 to 3.07) | 0.38 | 1.35 (0.69 to 2.66) |
| | | Weighted median | 441 | 0.96 | 1.01 (0.56 to 1.84) | 0.88 | 1.05 (0.58 to 1.90) |
| | | Inverse variance weighted | 441 | 1.00 | 1.00 (0.70 to 1.43) | 0.69 | 0.93 (0.65 to 1.33) |
| | | Simple mode | 441 | 0.53 | 0.65 (0.16 to 2.54) | 0.47 | 0.60 (0.15 to 2.40) |
| | | Weighted mode | 441 | 0.98 | 0.99 (0.45 to 2.19) | 0.91 | 1.05 (0.46 to 2.37) |
| CM | Head and Neck | MR Egger | 443 | 0.07 | 1.43 (0.97 to 2.11) | 0.24 | 1.26 (0.86 to 1.85) |
| | | Weighted median | 443 | 1.18E-02 | 1.52 (1.10 to 2.11) | 0.02 | 1.50 (1.07 to 2.10) |
| | | Inverse variance weighted | 443 | **9.67E-03** | **1.31 (1.07 to 1.61)** | **0.05** | **1.23 (1.00 to 1.50)** |
| | | Simple mode | 443 | 0.11 | 1.91 (0.87 to 4.17) | 0.11 | 1.95 (0.87 to 4.39) |
| | | Weighted mode | 443 | 0.25 | 1.33 (0.81 to 2.18) | 0.36 | 1.25 (0.78 to 2.00) |
| | Head and Neck (excl. Scalp) | MR Egger | 443 | 0.35 | 1.37 (0.71 to 2.63) | 0.66 | 1.15 (0.61 to 2.19) |
| | | Weighted median | 443 | 0.20 | 1.43 (0.83 to 2.45) | 0.39 | 1.29 (0.73 to 2.28) |
| | | Inverse variance weighted | 443 | **0.11** | **1.32 (0.94 to 1.87)** | **0.28** | **1.21 (0.86 to 1.69)** |
| | | Simple mode | 443 | 0.52 | 1.58 (0.40 to 6.30) | 0.54 | 1.55 (0.38 to 6.36) |
| | | Weighted mode | 443 | 0.35 | 1.43 (0.68 to 3.00) | 0.27 | 1.55 (0.71 to 3.38) |
| | Head and Neck (Scalp only) | MR Egger | 443 | 0.13 | 1.98 (0.81 to 4.83) | 0.52 | 1.34 (0.54 to 3.30) |
| | | Weighted median | 443 | 0.54 | 1.28 (0.57 to 2.87) | 0.69 | 1.18 (0.53 to 2.64) |
| | | Inverse variance weighted | 443 | **0.01** | **1.89 (1.18 to 3.03)** | **0.06** | **1.57 (0.98 to 2.54)** |
| | | Simple mode | 443 | 0.64 | 1.58 (0.23 to >10) | 0.64 | 1.58 (0.23 to 10.78) |
| | | Weighted mode | 443 | 0.86 | 1.11 (0.36 to 3.42) | 0.86 | 1.10 (0.38 to 3.22) |
| | Upper limb | MR Egger | 443 | 0.99 | 1.00 (0.68 to 1.46) | 0.50 | 0.88 (0.60 to 1.28) |
| | | Weighted median | 443 | 0.85 | 0.97 (0.70 to 1.35) | 0.46 | 0.88 (0.63 to 1.23) |
| | | Inverse variance weighted | 443 | 0.08 | 0.84 (0.68 to 1.02) | 0.02 | 0.79 (0.65 to 0.97) |
| | | Simple mode | 443 | 0.35 | 0.68 (0.31 to 1.51) | 0.14 | 0.55 (0.24 to 1.22) |
| | | Weighted mode | 443 | 0.91 | 0.98 (0.63 to 1.51) | 0.78 | 0.94 (0.60 to 1.46) |
| | Trunk | MR Egger | 443 | 0.92 | 0.99 (0.75 to 1.29) | 0.67 | 0.94 (0.72 to 1.24) |
| | | Weighted median | 443 | 0.93 | 0.99 (0.78 to 1.25) | 0.65 | 0.95 (0.75 to 1.20) |
| | | Inverse variance weighted | 443 | 0.30 | 0.93 (0.80 to 1.07) | 0.19 | 0.91 (0.79 to 1.05) |
| | | Simple mode | 443 | 0.61 | 0.85 (0.46 to 1.57) | 0.53 | 0.82 (0.44 to 1.54) |
| | | Weighted mode | 443 | 0.78 | 1.05 (0.73 to 1.53) | 0.97 | 0.99 (0.66 to 1.48) |
| | Lower limb | MR Egger | 443 | 0.57 | 1.13 (0.74 to 1.74) | 0.68 | 1.10 (0.71 to 1.69) |
| | | Weighted median | 443 | 0.27 | 0.81 (0.55 to 1.18) | 0.29 | 0.81 (0.56 to 1.19) |

| | | | | | | | |
|---|---|---|---|---|---|---|---|
| | | Inverse variance weighted | 443 | 0.22 | 0.87 (0.69 to 1.09) | 0.17 | 0.85 (0.68 to 1.07) |
| | | Simple mode | 443 | 0.43 | 0.69 (0.28 to 1.73) | 0.44 | 0.71 (0.29 to 1.72) |
| | | Weighted mode | 443 | 0.22 | 0.74 (0.46 to 1.20) | 0.26 | 0.76 (0.48 to 1.22) |

SNP-outliers here refer to the 4 detected MR-PRESSO SNP outliers (shown in Supplementary Table 11)

## Supplementary Table 20. Estimated MR ORs for per 1 SD change increase in genetically predicted MPB score on cutaneous melanoma risk stratified by (thick vs thin) Breslow thickness in the MIA dataset.

| Cancer | MR Method | Original estimate | | After removal of SNP-outliers | |
|---|---|---|---|---|---|
| | | Pvalue | OR | Pvalue | OR |
| Cutaneous melanoma (all thickness) | MR Egger | 0.90 | 1.02 (0.71 to 1.47) | 0.49 | 0.89 (0.63 to 1.25) |
| | Weighted median | 1.00 | 1.00 (0.75 to 1.34) | 0.92 | 0.98 (0.74 to 1.32) |
| | **Inverse variance weighted** | **0.52** | **1.07 (0.88 to 1.29)** | **0.88** | **0.99 (0.82 to 1.18)** |
| | Simple mode | 0.76 | 1.11 (0.56 to 2.19) | 0.65 | 1.17 (0.59 to 2.30) |
| | Weighted mode | 0.92 | 0.98 (0.64 to 1.49) | 0.93 | 0.98 (0.64 to 1.51) |
| Cutaneous melanoma (thick) | MR Egger | 0.85 | 1.04 (0.69 to 1.58) | 0.46 | 0.86 (0.58 to 1.27) |
| | Weighted median | 0.99 | 1.00 (0.71 to 1.41) | 0.73 | 0.94 (0.66 to 1.33) |
| | **Inverse variance weighted** | **0.33** | **1.12 (0.89 to 1.39)** | **0.90** | **1.01 (0.82 to 1.25)** |
| | Simple mode | 0.67 | 1.19 (0.54 to 2.59) | 0.65 | 1.21 (0.53 to 2.79) |
| | Weighted mode | 0.88 | 0.96 (0.59 to 1.58) | 0.94 | 0.98 (0.62 to 1.55) |
| Cutaneous melanoma (thin) | MR Egger | 0.82 | 1.05 (0.66 to 1.67) | 0.90 | 0.97 (0.62 to 1.53) |
| | Weighted median | 0.58 | 1.12 (0.76 to 1.65) | 0.93 | 1.02 (0.68 to 1.52) |
| | **Inverse variance weighted** | **0.73** | **1.04 (0.82 to 1.33)** | **0.96** | **0.99 (0.78 to 1.26)** |
| | Simple mode | 0.77 | 0.86 (0.32 to 2.36) | 0.81 | 0.88 (0.31 to 2.49) |
| | Weighted mode | 0.98 | 1.01 (0.57 to 1.76) | 0.83 | 0.94 (0.53 to 1.66) |

Definition of thick (Breslow thickness) melanoma=melanoma with >1mm Breslow thickness, thin (Breslow thickness) ≤1mm.

# Supplementary Figures

## Supplementary Figure 1. The anatomical definition for body site categories used in the present analyses

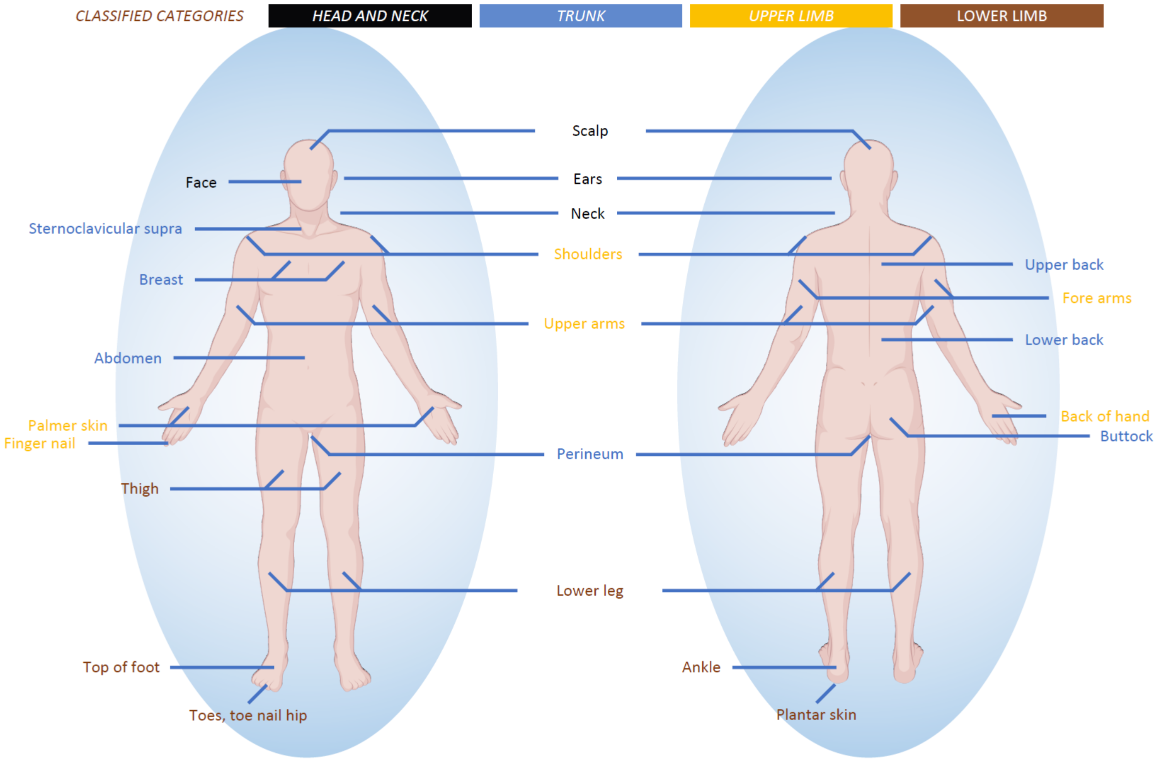


Note that the labeled body site regions are approximated.

**Supplementary Figure 2. Comparison of SNP effect sizes between the Ruth et al total testosterone GWAS and those derived from the UK Biobank (non-overlapping) subset.**

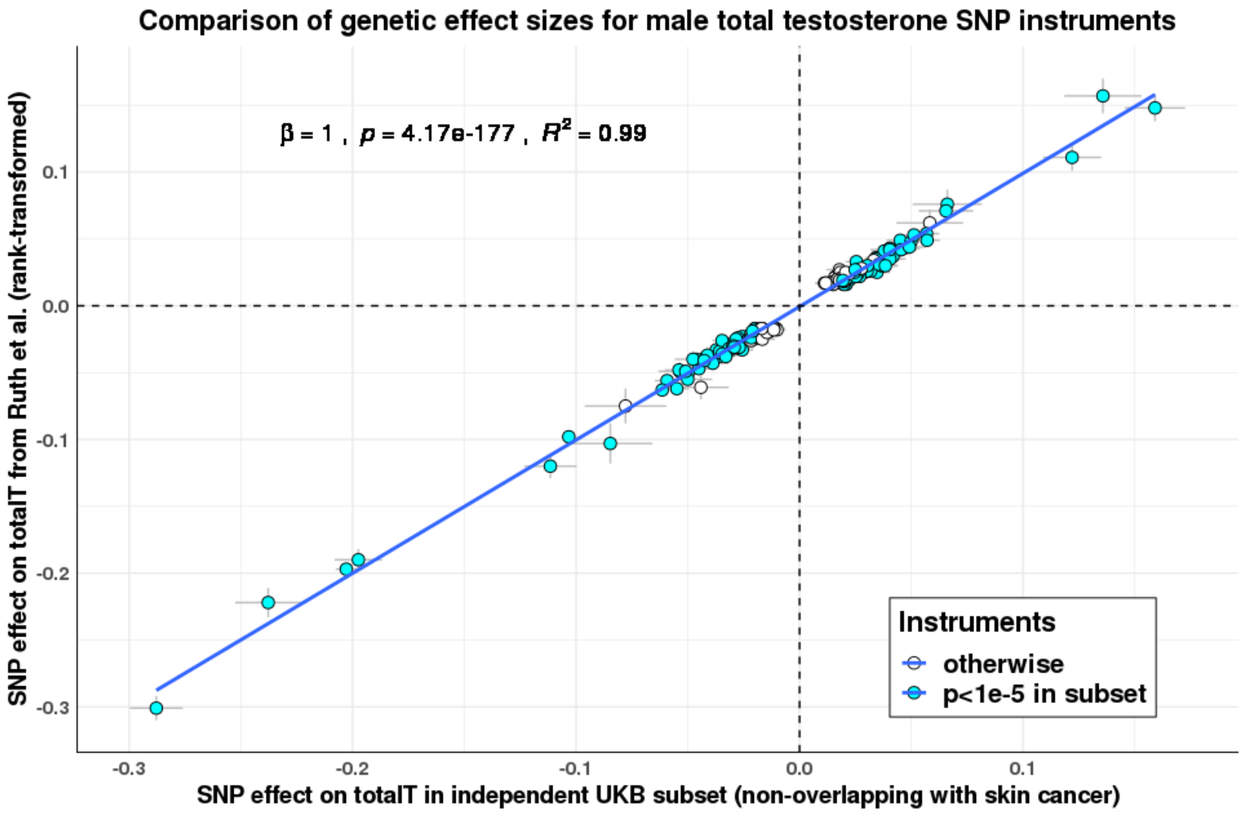


The number of independent SNPs associated with total testosterone evaluated in the regression above is 274. Error bars represent the standard error of the magnitude of association estimated in the respective GWASs. P-values are calculated from a two-tailed Z-test (on the regression estimate).

**Supplementary Figure 3. Comparison of SNP effect sizes between the Ruth et al free testosterone GWAS and those derived from the UK Biobank (non-overlapping) subset.**

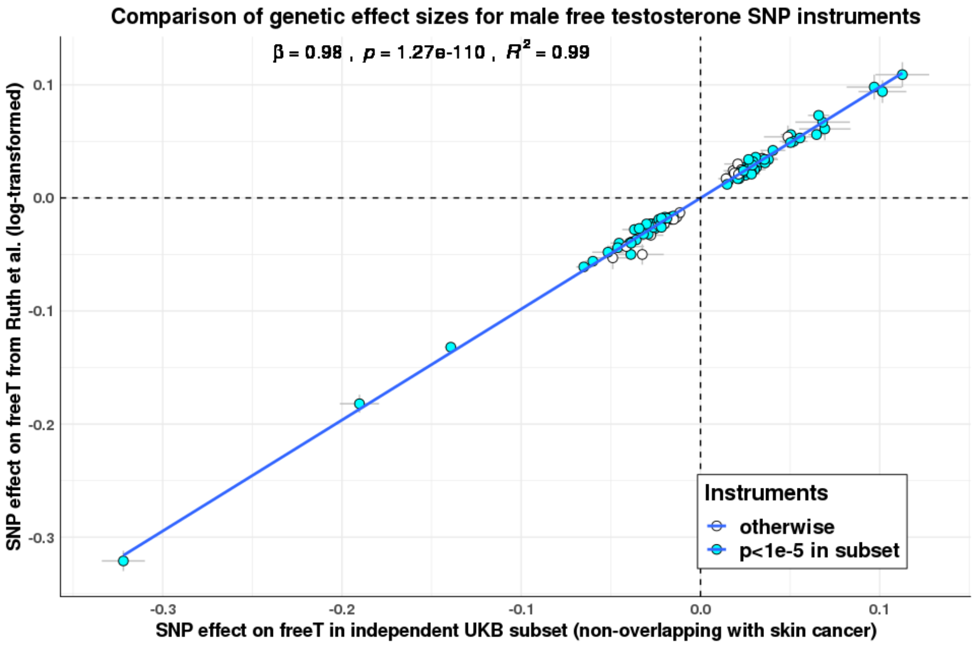


The number of independent SNPs associated with derived free testosterone evaluated in the regression above is 101. Error bars represent the standard errors of the magnitude of association estimated in the respective GWASs. P-values are calculated from a two-tailed Z-test (on the regression estimate).

**Supplementary Figure 4. Comparison of SNP effect sizes between the Ruth et al SHBG GWAS and those derived from the UK Biobank (non-overlapping) subset.**

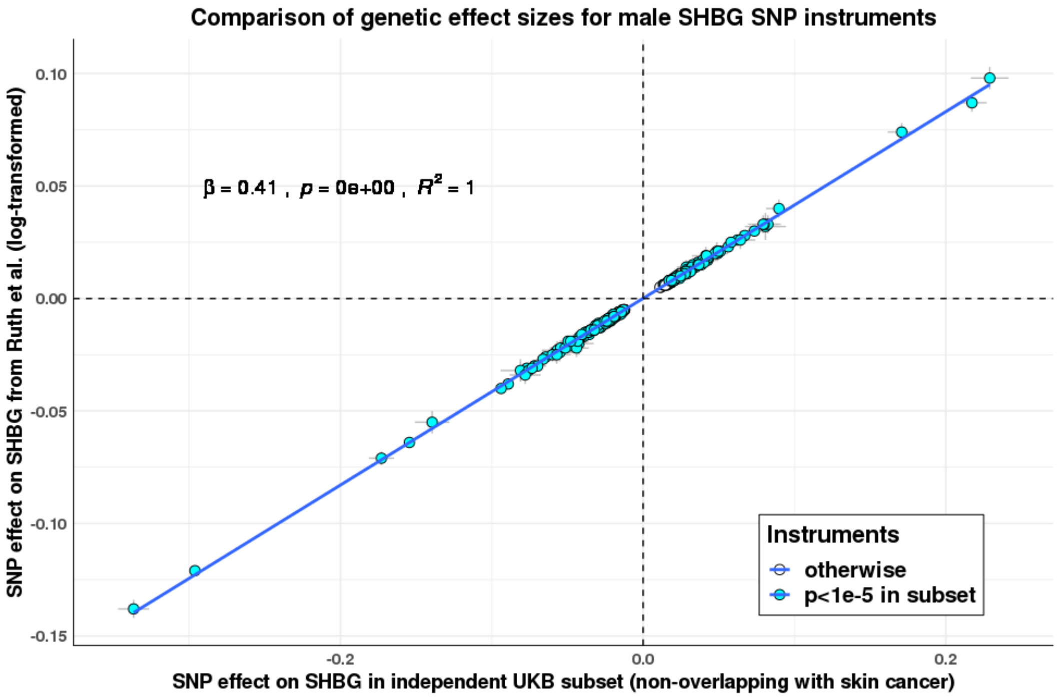


The number of independent SNPs associated with SHBG evaluated in the regression above is 473. Error bars represent the standard errors of the magnitude of association estimated in the respective GWASs. P-values are calculated from a two-tailed Z-test (on the regression estimate).

**Supplementary Figure 5. Estimated statistical power for MR analysis to detect plausible OR effect sizes in our present analysis.**

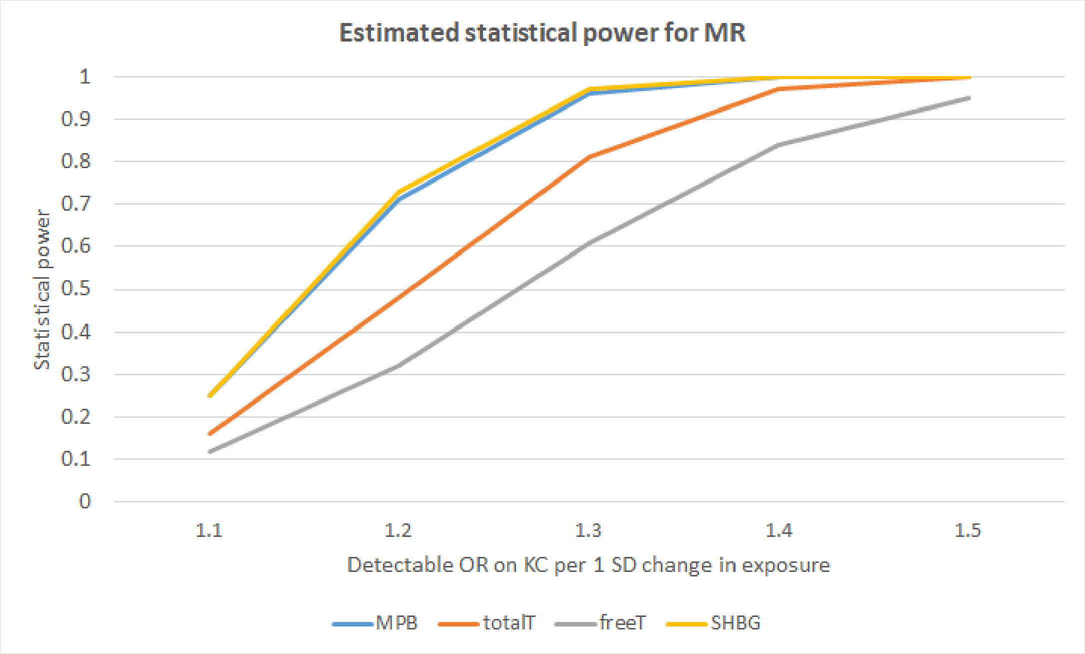


Power estimates were derived using the mRnd online MR power calculator based on estimated phenotypic variance explained by SNPs for each trait and sample size from the skin cancer GWASs.

## Supplementary Figure 6. MR funnel plots for the univariable MR association between MPB and BCC in the UK Biobank and QSKIN datasets

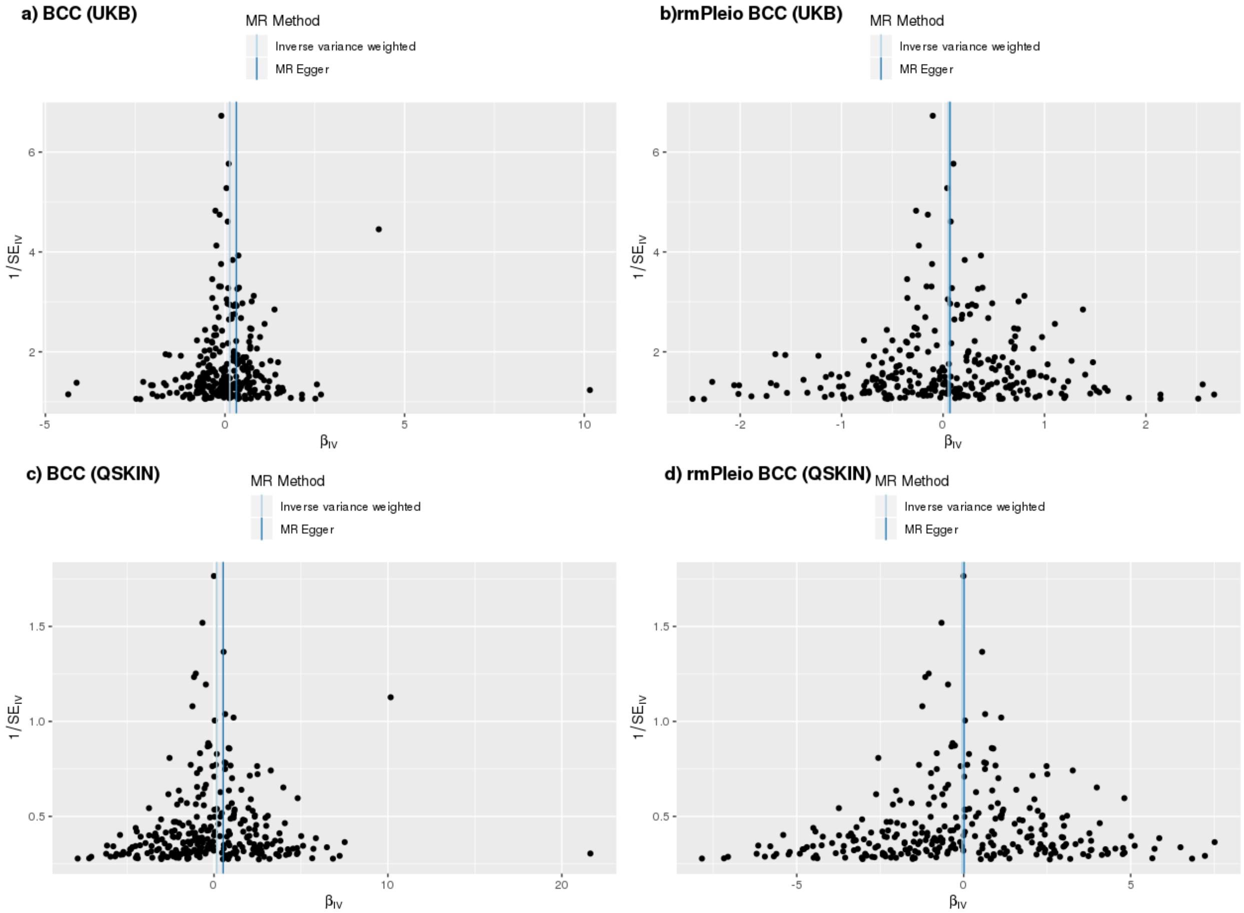


The label **rmPleio** refers to the trait-outcome MR association between MPB and the relevant skin cancer outcome after removing the pleiotropic SNP outliers from the analysis.

# Supplementary Figure 7. MR funnel plots for the univariable MR association between MPB and SCC in the UK Biobank and QSKIN datasets

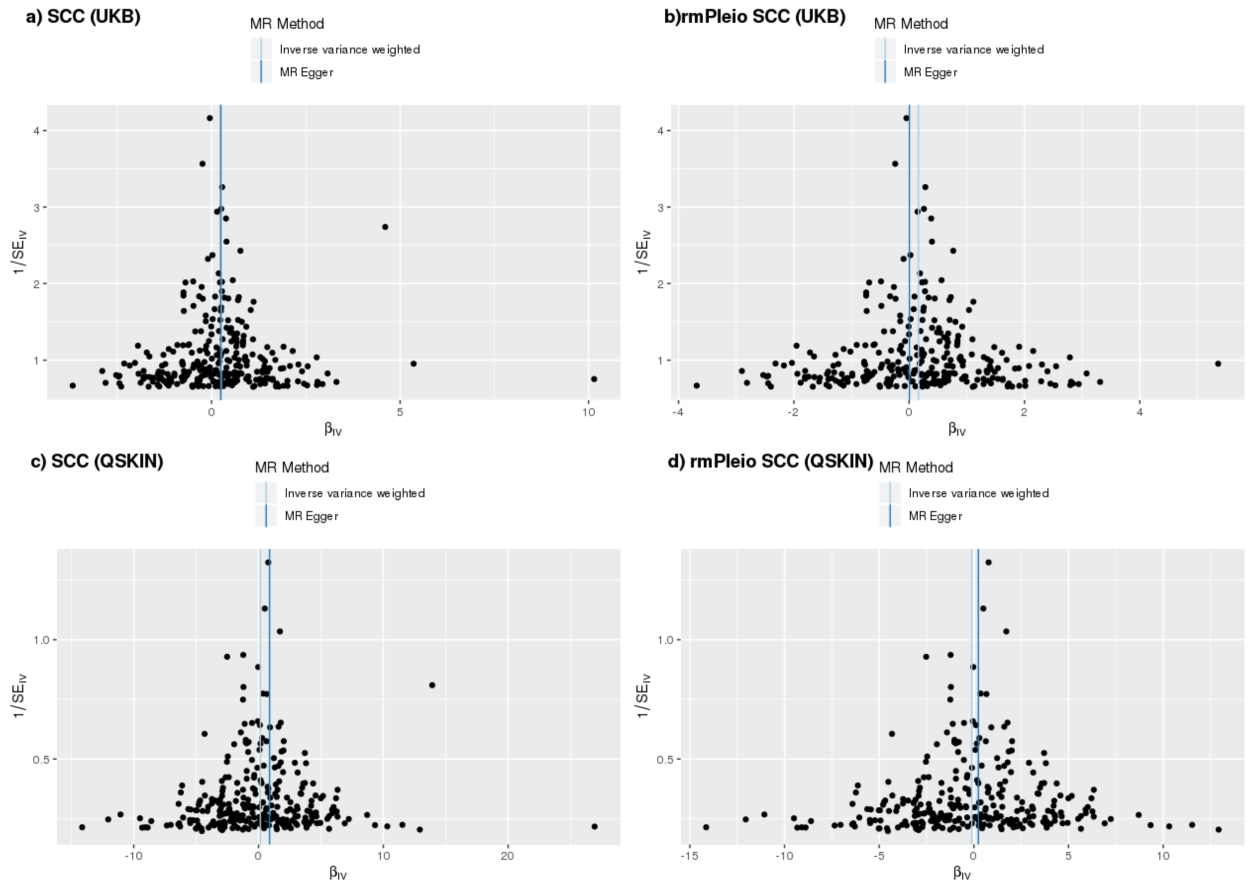


The label **rmPleio** refers to the trait-outcome MR association between MPB and the relevant skin cancer outcome after removing the pleiotropic SNP outliers from the analysis.

## Supplementary Figure 8. MR funnel plots for the univariable MR association between MPB and all KC in the UK Biobank and QSKIN datasets

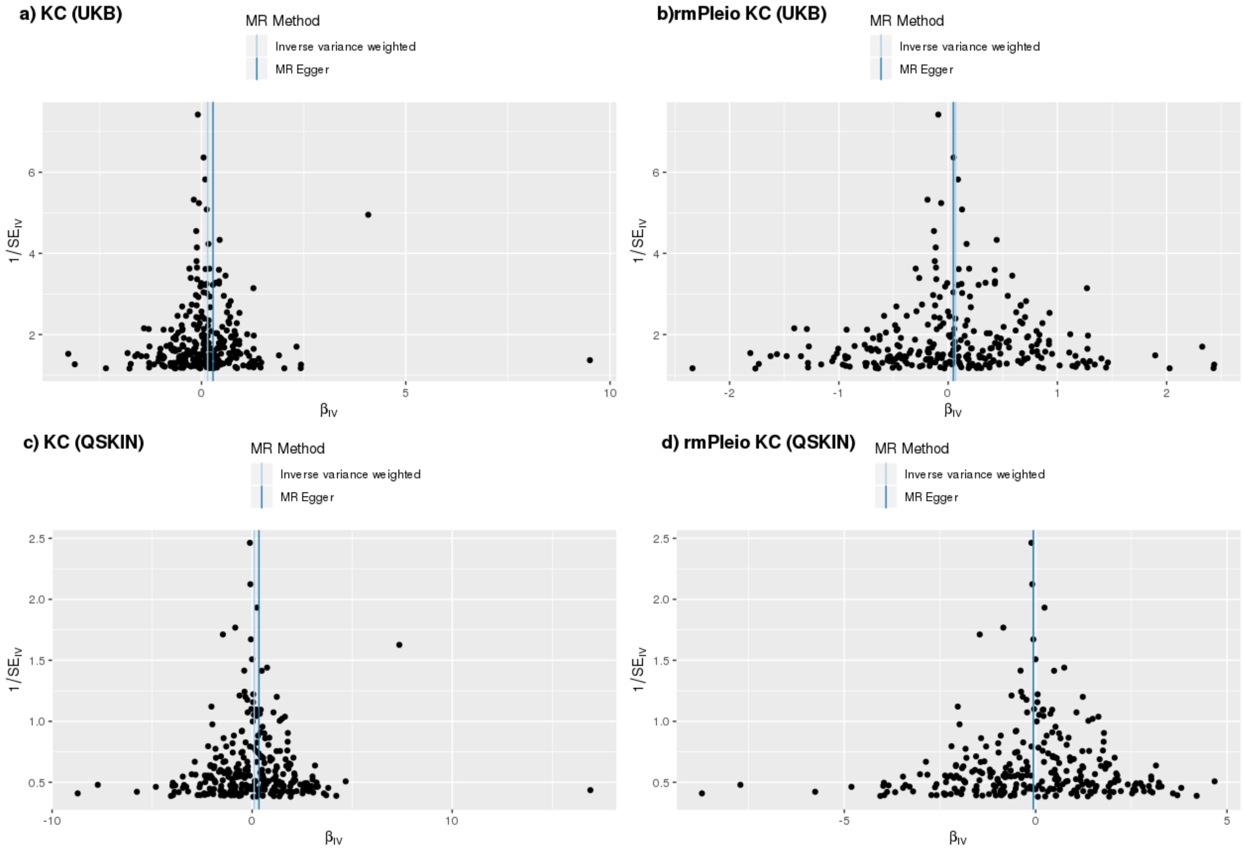


The label **rmPleio** refers to the trait-outcome MR association between MPB and the relevant skin cancer outcome after removing the pleiotropic SNP outliers from the analysis.

## Supplementary Figure 9. MR funnel plots for the univariable MR association between MPB and melanoma

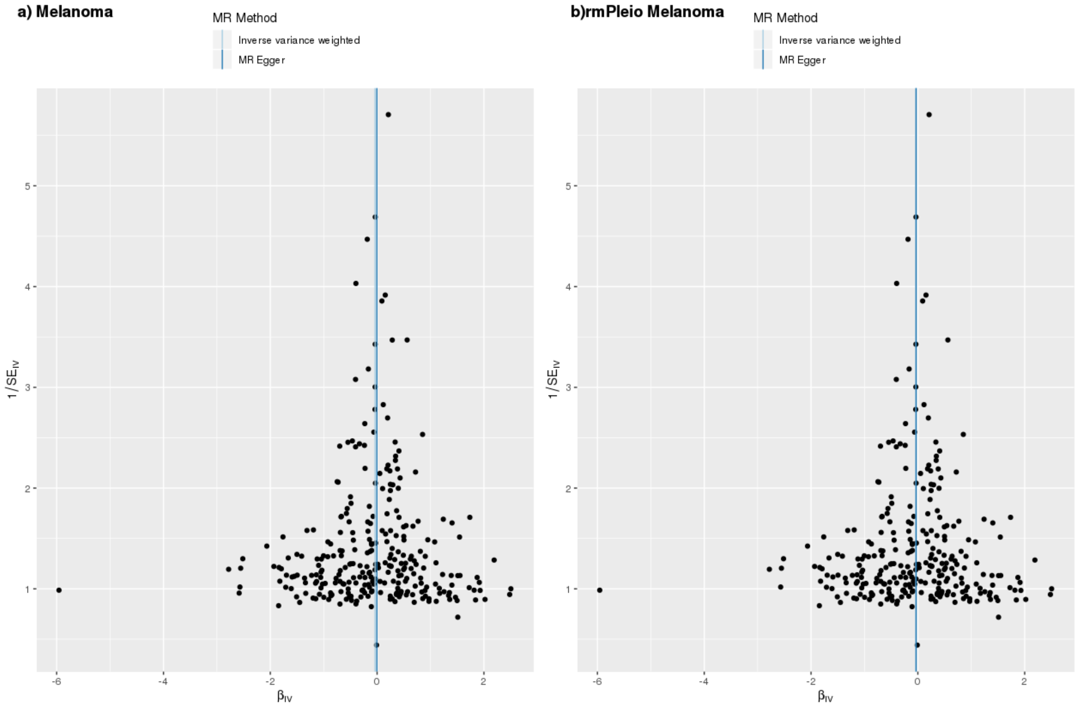


The label **rmPleio** refers to the trait-outcome MR association between MPB and the relevant skin cancer outcome after removing the pleiotropic SNP outliers from the analysis.

## Supplementary Figure 10. PheWAS plot for rs2669871

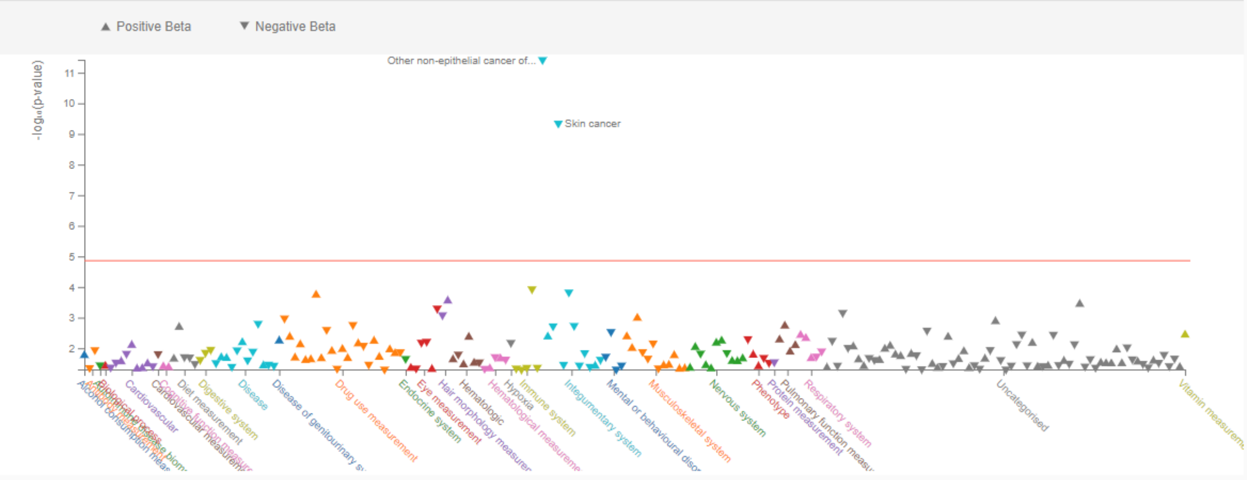


The y-axis of the plot represents the -log10 p-value of the association between the SNP and the relevant trait (in the x-axis) derived from GWAS data. The red bar refer to the (nominal) strength of association approximately at p-value=5x10e-5. The PheWAS lookup was performed by manually querying the SNP against the Open-target online platform (https://genetics.opentargets.org/). P-values are derived from a two-tailed Z-test on the association between the SNP and the relevant trait, unadjusted for multiple comparison.

## Supplementary Figure 11. PheWAS plot for rs3847069

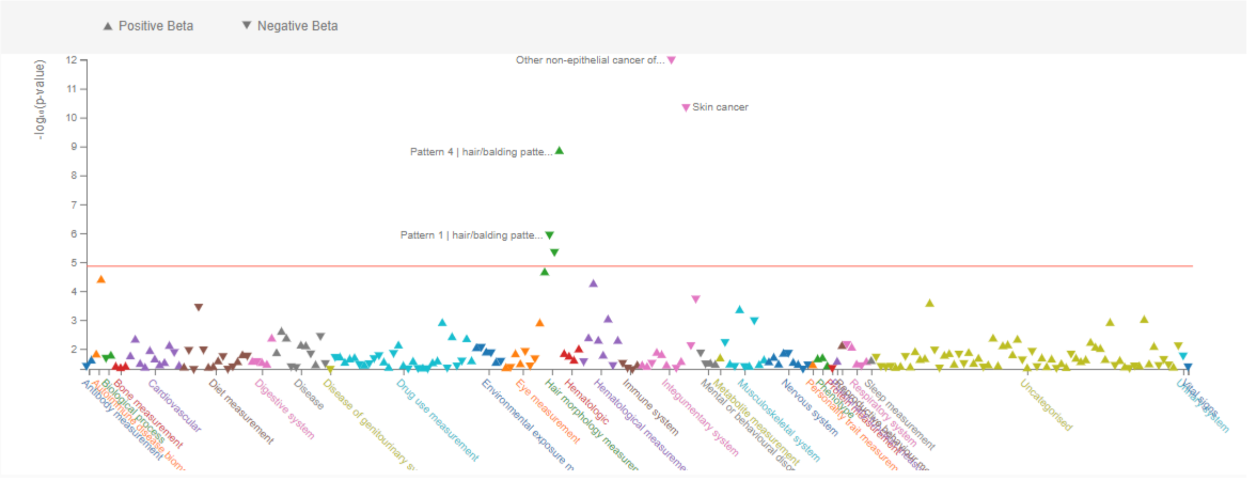


The y-axis of the plot represents the -log10 p-value of the association between the SNP and the relevant trait (in the x-axis) derived from GWAS data. The red bar refer to the (nominal) strength of association approximately at p-value=5x10e-5. The PheWAS lookup was performed by manually querying the SNP against the Open-target online platform (https://genetics.opentargets.org/). P-values are derived from a two-tailed Z-test on the association between the SNP and the relevant trait, unadjusted for multiple comparison.

## Supplementary Figure 12. PheWAS plot for rs1805007

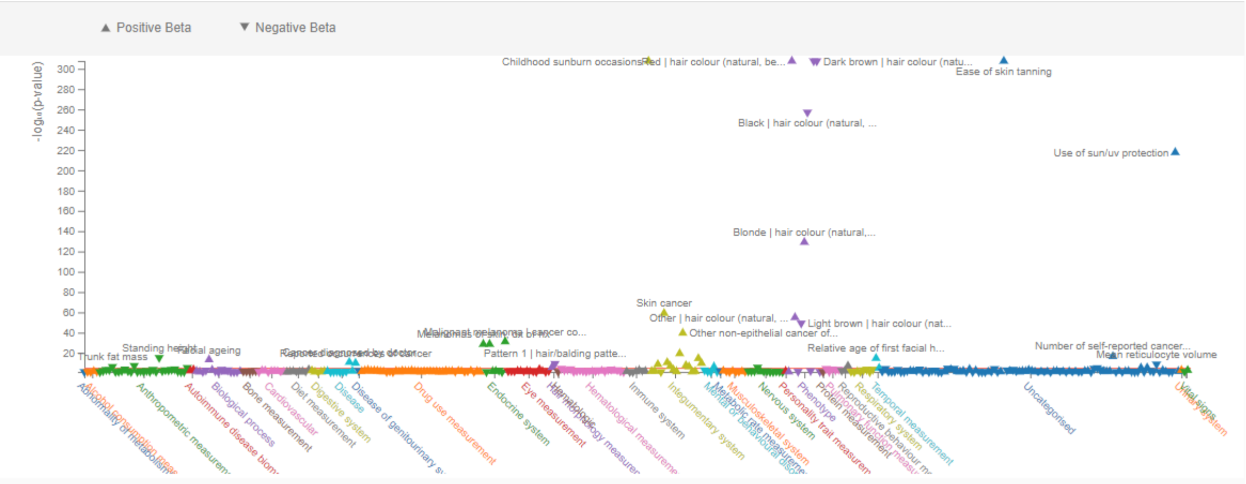


The y-axis of the plot represents the -log10 p-value of the association between the SNP and the relevant trait (in the x-axis) derived from GWAS data. The red bar refer to the (nominal) strength of association approximately at p-value=5x10e-5. The PheWAS lookup was performed by manually querying the SNP against the Open-target online platform (https://genetics.opentargets.org/).P-values are derived from a two-tailed Z-test on the association between the SNP and the relevant trait, unadjusted for multiple comparison.

## Supplementary Figure 13. PheWAS plot for rs12203592

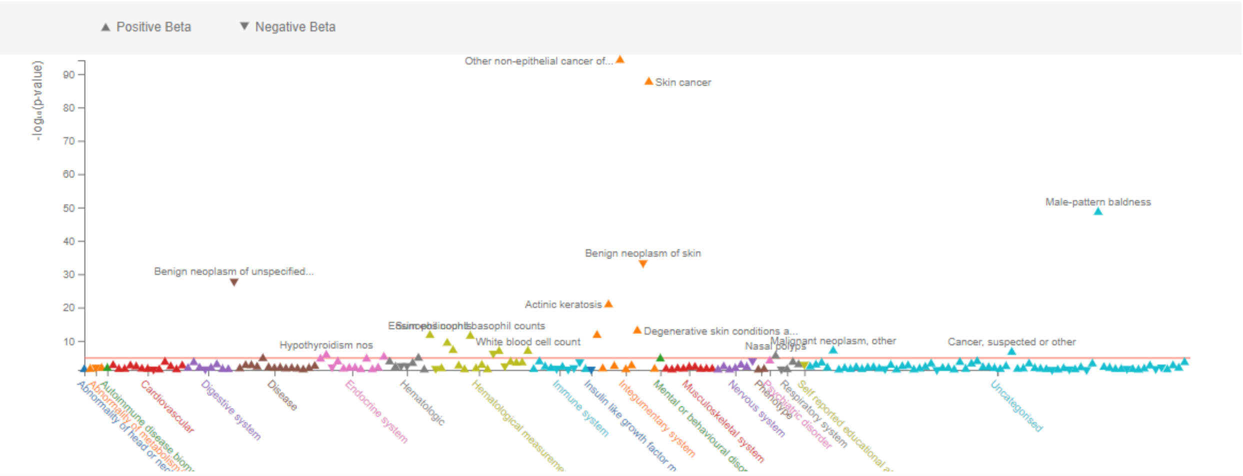


The y-axis of the plot represents the -log10 p-value of the association between the SNP and the relevant trait (in the x-axis) derived from GWAS data. The red bar refer to the (nominal) strength of association approximately at p-value=5x10e-5. The PheWAS lookup was performed by manually querying the SNP against the Open-target online platform (https://genetics.opentargets.org/). P-values are derived from a two-tailed Z-test on the association between the SNP and the relevant trait, unadjusted for multiple comparison.

## Reference


1. Landi, M. T. *et al.* Genome-wide association meta-analyses combining multiple risk phenotypes provide insights into the genetic architecture of cutaneous melanoma susceptibility. *Nat. Genet.* **52**, 494–504 (2020).
2. Olsen, C. M. *et al.* Cohort profile: The QSkin Sun and Health Study. *International Journal of Epidemiology* vol. 41 929–929i Preprint at https://doi.org/10.1093/ije/dys107 (2012).
3. Liyanage, U. E. *et al.* Combined analysis of keratinocyte cancers identifies novel genome-wide loci. *Hum. Mol. Genet.* **28**, 3148–3160 (2019).
4. Byrne, E. M. *et al.* Cohort profile: the Australian genetics of depression study. *BMJ Open* **10**, e032580 (2020).
5. Keung, E. Z. & Gershenwald, J. E. The eighth edition American Joint Committee on Cancer (AJCC) melanoma staging system: implications for melanoma treatment and care. *Expert Rev. Anticancer Ther.* **18**, (2018).
6. Vermeulen, A., Verdonck, L. & Kaufman, J. M. A critical evaluation of simple methods for the estimation of free testosterone in serum. *J. Clin. Endocrinol. Metab.* **84**, 3666–3672 (1999).
7. Verbanck, M., Chen, C.-Y., Neale, B. & Do, R. Detection of widespread horizontal pleiotropy in causal relationships inferred from Mendelian randomization between complex traits and diseases. *Nat. Genet.* **50**, 693–698 (2018).
8. Canela-Xandri, O., Rawlik, K. & Tenesa, A. An atlas of genetic associations in UK Biobank. *Nat. Genet.* **50**, 1593–1599 (2018).

9. Ghoussaini, M. *et al.* Open Targets Genetics: systematic identification of trait-associated genes using large-scale genetics and functional genomics. *Nucleic Acids Res.* **49**, D1311–D1320 (2021).

10. Cho, Y. *et al.* Exploiting horizontal pleiotropy to search for causal pathways within a Mendelian randomization framework. *Nat. Commun.* **11**, 1010 (2020).

11. Davies, N. M., Holmes, M. V. & Davey Smith, G. Reading Mendelian randomisation studies: a guide, glossary, and checklist for clinicians. *BMJ* **362**, k601 (2018).

12. Ong, J.-S. *et al.* A comprehensive re-assessment of the association between vitamin D and cancer susceptibility using Mendelian randomization. *Nat. Commun.* **12**, 246 (2021).

13. Yavorska, O. O. & Burgess, S. MendelianRandomization: an R package for performing Mendelian randomization analyses using summarized data. *Int. J. Epidemiol.* **46**, 1734–1739 (2017).

14. Sanderson, E., Spiller, W. & Bowden, J. Testing and correcting for weak and pleiotropic instruments in two-sample multivariable Mendelian randomization. *Stat. Med.* **40**, 5434–5452 (2021).

15. Ruth, K. S. *et al.* Genome-wide association study with 1000 genomes imputation identifies signals for nine sex hormone-related phenotypes. *Eur. J. Hum. Genet.* **24**, 284–290 (2016).

----------------------------------------END OF DOCUMENT ----------------------------------------------------------
