# Supplementary material for: Uncovering the complex relationship between balding, testosterone and skin cancers in men
Source: Nat Commun. 2023 Oct 3;14:5962. doi: 10.1038/s41467-023-41231-8 (PMC10547720; doi:10.1038/s41467-023-41231-8)
Supplement: Supplementary file 3 — Description of Additional Supplementary Files [file 41467_2023_41231_MOESM3_ESM.pdf]

Title: Supplementary Data 1

Description: Genetic summary data for the relevant SNP instruments on each exposure (risk factor), and the summary results from alternative univariable MR models.
