# Supplementary material for: Uncovering the complex relationship between balding, testosterone and skin cancers in men
Source: Nat Commun. 2023 Oct 3;14:5962. doi: 10.1038/s41467-023-41231-8 (PMC10547720; doi:10.1038/s41467-023-41231-8)
Supplement: Supplementary file 5 — Reporting Summary [file 41467_2023_41231_MOESM5_ESM.pdf]

nature portfolio

Corresponding author(s): Jue-Sheng Ong

Last updated by author(s): Jul 27, 2023

Reporting Summary

Nature Portfolio wishes to improve the reproducibility of the work that we publish. This form provides structure for consistency and transparency in reporting. For further information on Nature Portfolio policies, see our Editorial Policies and the Editorial Policy Checklist.

Statistics

For all statistical analyses, confirm that the following items are present in the figure legend, table legend, main text, or Methods section.

n/a	Confirmed	
☐	☒	The exact sample size (*n*) for each experimental group/condition, given as a discrete number and unit of measurement
☐	☒	A statement on whether measurements were taken from distinct samples or whether the same sample was measured repeatedly
☐	☒	The statistical test(s) used AND whether they are one- or two-sided<br>*Only common tests should be described solely by name; describe more complex techniques in the Methods section.*
☐	☒	A description of all covariates tested
☐	☒	A description of any assumptions or corrections, such as tests of normality and adjustment for multiple comparisons
☐	☒	A full description of the statistical parameters including central tendency (e.g. means) or other basic estimates (e.g. regression coefficient) AND variation (e.g. standard deviation) or associated estimates of uncertainty (e.g. confidence intervals)
☐	☒	For null hypothesis testing, the test statistic (e.g. *F*, *t*, *r*) with confidence intervals, effect sizes, degrees of freedom and *P* value noted<br>*Give P values as exact values whenever suitable.*
☒	☐	For Bayesian analysis, information on the choice of priors and Markov chain Monte Carlo settings
☒	☐	For hierarchical and complex designs, identification of the appropriate level for tests and full reporting of outcomes
☐	☒	Estimates of effect sizes (e.g. Cohen's *d*, Pearson's *r*), indicating how they were calculated

Our web collection on statistics for biologists contains articles on many of the points above.

Software and code

Policy information about availability of computer code

Data collection	Data for the phenotypes involved in the study were obtained from the UK Biobank.<br>Data source for GWAS summary statistics for individual skin cancers had been provided in Supplementary information.<br>No specialist software was used for data collection.
Data analysis	The GWAS analysis for sex hormones and male pattern baldness were performed using BOLT-LMM v2.3 (available in http://data.broadinstitute.org/alkesgroup/BOLT-LMM/). Association analysis performed in open-source statistical software R v4.0.2 (https://www.r-project.org/) using the TwoSample Mendelian randomization v0.4.22 R package (https://github.com/MRCIEU/TwoSampleMR) and the MendelianRandomization v0.5.0 R package. Illustrations produced using the ggplot2 v3.2.1 R package available from the R CRAN repository (https://cran.r-project.org/).<br>A test example for our MR analysis codes used for the analysis have been supplied in the Zenodo code repository (DOI: 10.5281/zenodo.7988335).

For manuscripts utilizing custom algorithms or software that are central to the research but not yet described in published literature, software must be made available to editors and reviewers. We strongly encourage code deposition in a community repository (e.g. GitHub). See the Nature Portfolio guidelines for submitting code & software for further information.

Data

Policy information about availability of data

All manuscripts must include a data availability statement. This statement should provide the following information, where applicable:

- Accession codes, unique identifiers, or web links for publicly available datasets
- A description of any restrictions on data availability
- For clinical datasets or third party data, please ensure that the statement adheres to our policy

The genetic summary data for the risk factors presented in this manuscript are primarily derived from the UK Biobank cohort (Supplementary Data 1). The UK Biobank phenotype and genetic data can be obtained by application directly to the UK Biobank. The sex-specific melanoma GWAS meta-analysis summary statistics can be obtained through direct application to the study principal investigators (M. H. Law matthew.law@qimrberhofer.edu.au; M. M. Iles M.M.Iles@leeds.ac.uk; M. T. Landi landim@mail.nih.gov). The complete GWAS summary statistics data for KC derived from the QSkin study is available under restricted access, and the data access can be obtained by contacting the following QSkin principal investigators (D. C. Whiteman David.Whiteman@qimrberghofer.edu.au; C M. Olsen Catherine.Olsen@qimrberghofer.edu.au). The GWAS findings from the site-stratified melanoma data in the MIA is available under restricted access, and data access can be obtained by contacting the MIA investigator R.A. Scolyer (EAscolyer@melanoma.org.au). The summary statistics for SNPs analysed in this study are also provided in Supplementary Data 1. Source data are provided with this paper.

Human research participants

Policy information about studies involving human research participants and Sex and Gender in Research.

Reporting on sex and gender	This study is designed to only analyse pattern baldness and risk of skin cancer in men (hence the term male-pattern baldness). The recruitment and identification of male participants were carried out by individual studies from the GenoMEL consortium, of which the present study only relied on summary level genetic data. For detailed information on how sex/ gender is defined in those studies, please refer to the respective publications (data source) referenced in the main text. For the UK Biobank analysis, we relied on genetic-sex inferred directly from participant genotype data.
Population characteristics	The population consists primarily of middle aged male participants, of white European ancestry. Participants for this study is not selected based on genotypes, age (>18 years of age), other disease status. The GWAS analysis on skin cancers were performed based on clinically confirmed skin cancer diagnosis status, with age-matched healthy controls.
Recruitment	This study relies on summary level genetic data for analysis and does not involve direct recruitment of study participants. The study recruitment for the QSKIN cohort, AGDS, and the Melanoma Institute of Australia (MIA) studies have been reported elsewhere (please see main text for references).
Ethics oversight	The study protocol is approved by the QIMRB-Human Research Ethics Committee (HREC). Please refer to the acknowledgment section for ethics approval of the GenoMEL GWAS study.

Note that full information on the approval of the study protocol must also be provided in the manuscript.

Field-specific reporting

Please select the one below that is the best fit for your research. If you are not sure, read the appropriate sections before making your selection.

☒ Life sciences ☐ Behavioural & social sciences ☐ Ecological, evolutionary & environmental sciences

For a reference copy of the document with all sections, see nature.com/documents/nr-reporting-summary-flat.pdf

Life sciences study design

All studies must disclose on these points even when the disclosure is negative.

Sample size	The risk factor GWASs varied by traits (sex hormones, pattern baldness, pigmentation) while the cancer GWAS sample size varies by skin cancer type (see Methods section from main manuscript for full description of individual sample sizes). Please also see supplementary tables for the breakdown of cases by anatomical body sites. Sample size for the MR study was determined based on power calculation (the minimum sample size to achieve 80% power for detecting an OR of 1.2 or more for a 1 SD change in the risk factor).
Data exclusions	Genetic instruments below MAF of 0.01 and with an association pvalue > 5e-8 with their respective risk factor were excluded.
Replication	Results replicated with alternative instrument sets and instruments from GWAS studies using more stringent SNP-association criteria. Estimates derived from the QSKIN, UK Biobank and MIA datasets are all independent (experiments performed independently).
Randomization	Randomization achieved by study design (where variants inherited at random can mimic a natural randomization of each risk factor: pattern baldness, endogenous sex hormone levels, pigmentation variables; based on Mendel's Law of independent assortment)
Blinding	Information on specific genotype unlikely made aware to subjects, nor will the subject's knowledge on their genotype(s) result in changes on

behaviour associated with cancer outcome, at a scale substantial enough to generate bias. Moreover, study approach only utilises summary level data for inference. Due to the nature of the genetic study, investigators are also blinded to the allocation of genetically high risk and low risk (skin cancer) patient groups.

Reporting for specific materials, systems and methods

We require information from authors about some types of materials, experimental systems and methods used in many studies. Here, indicate whether each material, system or method listed is relevant to your study. If you are not sure if a list item applies to your research, read the appropriate section before selecting a response.

Materials & experimental systems

n/a	Involved in the study	
☒	☐	Antibodies
☒	☐	Eukaryotic cell lines
☒	☐	Palaeontology and archaeology
☒	☐	Animals and other organisms
☒	☐	Clinical data
☒	☐	Dual use research of concern

Methods

n/a	Involved in the study	
☒	☐	ChIP-seq
☒	☐	Flow cytometry
☒	☐	MRI-based neuroimaging
